# STING signaling pathway: An oasis in the glioblastoma immune desert

**DOI:** 10.7150/thno.128135

**Published:** 2026-01-01

**Authors:** Yafei Wang, Weichen Duan, Chuang Yan, Xuenan Li, Rui Xing, Li Yu, Jiajia Chen

**Affiliations:** Department of Oncology, Shengjing Hospital of China Medical University, Shenyang, 110004, China.

**Keywords:** glioma, STING signaling pathway, tumor microenvironment, STING agonists, immunotherapy

## Abstract

Glioblastoma (GBM) is an “immune desert” tumor, characterized by a highly immunosuppressive tumor microenvironment (TME), which leads to immune evasion and resistance to immunotherapies. The stimulator of interferon (IFN) genes (STING) signaling pathway serves as a central hub for priming anti-tumor immunity by driving the production of type I IFNs. Thus, STING activation has shown promise for overcoming immunosuppressive TME and inhibiting tumor malignancies. Accumulating preclinical evidence shows that STING agonists exert strong antitumor effects across multiple GBM models. However, the diverse and complex roles of the STING signaling pathway in reshaping the GBM microenvironment have not been fully summarized or elucidated. This review provides an overview of the mechanisms underlying STING dysregulation and the regulatory effect of STING activation on immune cell infiltration, priming, and function. Moreover, STING agonist monotherapies, and their combination regimens or delivery via innovative platforms for GBM treatment, are critically appraised, highlighting their implications for future clinical translation.

## 1. Introduction

Glioma, arising from glial cells, is the most common primary brain tumor of the central nervous system (CNS). In the World Health Organization (WHO) classification (I-IV), grade IV glioma glioblastoma (GBM) is the most malignant form 1. The treatment of GBM is challenged by its diffuse infiltration, high heterogeneity, treatment resistance, and rapid recurrence 1, 2. Despite standard therapies, including surgery, radiotherapy, and temozolomide (TMZ)-based chemotherapy, GBM still has a dismal prognosis with a 5-year survival rate less than 10% 3. Thus, more efforts are urgently needed to explore the pathological features of GBM and identify novel therapeutic targets.

It is generally accepted that the dysregulated immune environment contributes to the progression, invasion, and therapeutic resistance of GBM. GBM is traditionally considered an “immune cold” tumor, characterized by a highly immunosuppressive tumor microenvironment (TME). The TME involves few lymphocytic infiltrations, a predominance of immunosuppressive cells, elevated immune checkpoint molecules, and numerous immune-inhibitory cytokines 4. This highly immunosuppressive TME facilitates immune evasion and drives resistance to various immunotherapies, such as anti-PD-1/PD-L1 therapies and CTLA-4 blockade. To overcome these challenges, recent research is increasingly focused on exploring innovative immunomodulatory mechanisms, adjuvants, and agonists. The stimulator of interferon (IFN) genes (STING, also known as MITA or ERIS) is currently well-studied and being evaluated as a compelling target for anti-GBM immunity 5. STING functions as a key hub linking cytosolic DNA to innate immune response. Upon binding double-stranded DNA (dsDNA), cyclic GMP-AMP synthase (cGAS) undergoes allosteric activation and catalyzes the production of 2' 3'-cyclic GMP-AMP (cGAMP), a second messenger that activates STING. The activated STING subsequently triggers the release of IFNs and various proinflammatory cytokines, driving immune regulation 6. In GBM, STING activation can amplify type I IFN-mediated immune loops, reverse the suppressive properties of myeloid-derived suppressor cells (MDSCs) and macrophages, facilitate dendritic cell (DC) maturation and antigen presentation, and stimulate T cell immunity 5, 7.

Given its potential to overcome the immunosuppressive TME, STING is emerging as a promising target to combat GBM. However, the regulatory mechanisms of STING and its precise effects on GBM TME remodeling have not been thoroughly summarized. This review aims to summarize the immune-modulatory functions of the STING signaling pathway across various cell types within the TME in GBM. The mechanisms underlying STING dysregulation and the therapeutic potential of STING agonists are also summarized to broaden GBM treatment options. Unlike other published reviews in GBM, this review focused on the role of the STING signaling pathway in overcoming immunosuppression in GBM.

## 2. The biological role of the STING signaling pathway

**Figure 1:**
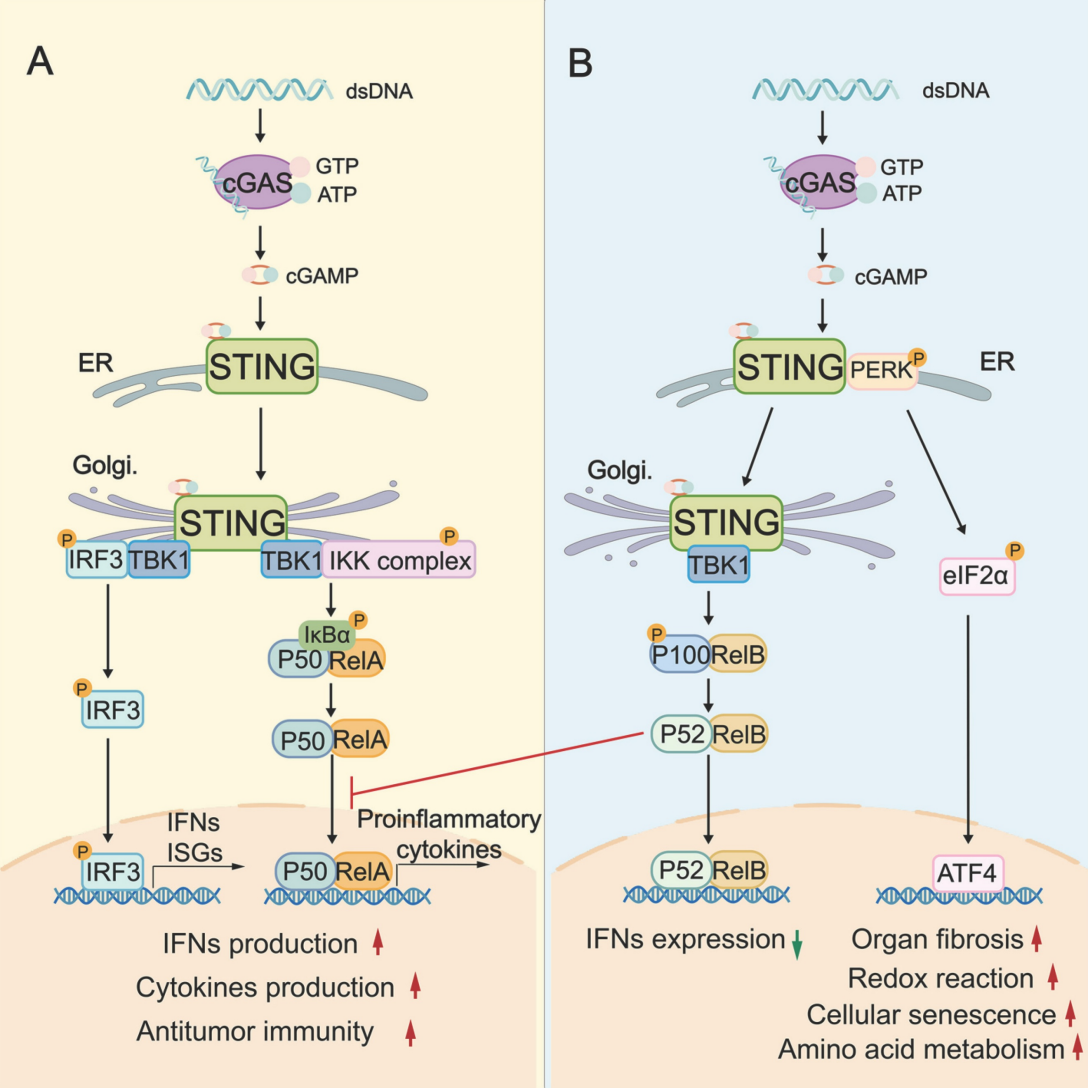
** The biological role of the cGAS-STING signaling pathway. A:** The canonical STING signaling pathway. cGAS binds to cytosolic dsDNA, leading to the synthesis of cGAMP. cGAMP then binds and activates STING, triggering the trafficking of STING from ER to Golgi, where it assembles the TBK1-IRF3 and IKK complexes. TBK1-phosphorylated IRF3 induces type I IFNs/ISGs, while the IKK-NF-κB (RelA/p50) axis promotes proinflammatory cytokine transcription, thereby supporting antitumor immunity. **B:** The “non-canonical” STING signaling pathway. cGAMP additionally activates a non-canonical STING-PERK-eIF2α pathway, wherein STING activates the ER stress kinase PERK. PERK activation leads to eIF2α phosphorylation, which enables translation of specific mRNAs that encode ATF4. Besides, STING activation triggers the non-canonical NF-κB (RelB/p52) signaling pathway, which in turn prevents RelA recruitment. These non-canonical pathways are critical for redox reactions, amino acid metabolism, organ fibrosis, and cellular senescence. cGAS: cyclic GMP-AMP synthase; dsDNA: double-stranded DNA; ER: endoplasmic reticulum; Golgi: Golgi apparatus; IKK complex: IκB kinase complex; TBK1: TANK-binding kinase 1; IRF3: interferon regulatory factor 3; ISGs: IFN-stimulated genes; PERK: protein kinase RNA-like ER kinase; ATF4: activating transcription factor 4.

The canonical STING signaling pathway plays a pivotal role in stimulating IFN-mediated immune response. This activation begins when cGAS binds to cytosolic dsDNA, leading to the synthesis of the second messenger cGAMP. cGAMP then binds to STING and induces a conformational change in STING, and promotes STING translocation from the endoplasmic reticulum (ER) to the ER-Golgi intermediate compartments and Golgi. This activated STING then recruits TANK-binding kinase 1 (TBK1) and interferon regulatory factor 3 (IRF3) to the Golgi, where TBK1 phosphorylates the C-terminus of IRF3. Phosphorylated IRF3 dimerizes, translocates to the nucleus, and drives the transcription of type I IFNs and various IFN-stimulated genes (ISGs) 8-10 (Figure 1). Concurrently, STING can activate the canonical NF-κB pathway by recruiting the IκB kinase (IKK) complex. The IKK complex phosphorylates and degrades IκB proteins, releasing NF-κB subunits RelA and p50 to translocate into the nucleus, where they promote transcription of proinflammatory cytokines such as IL-6 and TNF-α 11-13 (Figure 1). To control the intensity and duration of STING signaling pathway, activated STING can be trafficked into endolysosomes for lysosomal degradation or sorted into recycling endosomes for ESCRT-dependent microautophagy 14.

While the canonical STING signaling pathway is well-characterized, alternative “non-canonical” STING signaling pathways, which are mainly independent of the STING-TBK1-IRF3 axis, are increasingly recognized. At the ER, cGAMP can additionally activate a non-canonical STING-PERK-eIF2α pathway. In this axis, STING directly activates the ER stress kinase PERK (protein kinase RNA-like ER kinase), leading to eIF2α (eukaryotic initiation factor 2α) phosphorylation and broad translational reprogramming. This pathway is independent of the unfolded protein response and prior to TBK1-IRF3 activation (Figure 1) 15. RelB/p52 NF-κB signaling is another non-canonical pathway (Figure 1), which can be preferentially stimulated in DCs. Interestingly, the nuclear translocation of RelB/p52 has been observed to inhibit IFN-β transcription driven by canonical RelA axis 16. It is plausible that there is competitive crosstalk between canonical and noncanonical NF-κB pathways, which might disturb canonical STING-IFN-mediated immune regulation (Figure 1).

The STING signaling pathway is critical in the biological processes of antiviral defense, inflammatory modulation, and protection against tumorigenesis. Most studies focused on the canonical STING signaling pathway. In the antiviral aspect, once activated by virus-derived DNA, the STING signaling pathway triggers TBK1/IRF3- and NF-κB-dependent proinflammatory responses, releasing inflammatory cytokines (e.g., IL-6, TNF), chemokines (e.g., CXCL10, CCL5), and ISGs (e.g., ISG15, MX1) 17 to restrict viral replication. Besides, STING can stabilize transketolase in the pentose phosphate pathway by reducing ubiquitination in host cells. This process is essential for metabolic support for antiviral immunity 18. In tumors, STING plays a vital role in regulating IFN-dependent immune cell remodeling and immune responses. Genomic instability and cellular stress lead to the accumulation of cytosolic DNA and cGAMP in tumors. These cGAMP or external agonists activate the STING signaling pathway in both neoplastic cells and neighboring cells (e.g., DCs, macrophages, endothelial cells), eliciting a potent type I IFN and chemokine/cytokine-mediated innate immune cell recruitment and priming. Type I IFNs further bridge innate immune activation with T cell-mediated adaptive immunity by enhancing antigen presentation of antigen-presenting cells (APCs). Consequently, these STING-driven responses reshape the immune landscape, enhance immune surveillance, and promote tumor regression and durable immune memory 5, 19, 20. STING activation is increasingly being explored to overcome immunosuppression and therapy resistance. For example, STING agonist-lipid nanoparticles increased the expression of CD3, CD4, NK1.1, PD-1, and IFN-γ in TME. These changes enhanced NK (natural killer) cell-mediated tumor-killing, converted immunologically cold tumors into hot ones, and ultimately resensitized the melanoma model to immune checkpoint inhibition 21. In ovarian cancer, resistance to PARP inhibitors was associated with an expanded population of protumor macrophages driven by hyperactivated STAT3 signaling. However, STING activation inhibited protumor genes (Fn1, Plxdc2, Tgfb2), and concurrently upregulated antitumor genes (Ccl5, Ly6a, Ly6i) in macrophages. It shifted macrophages towards the pro-inflammatory subtype that overcame the PARP inhibitor resistance 22. Collectively, these findings underscore the STING-IFNs axis as a promising target for initiating and enhancing innate/adaptive immunity. Moreover, non-canonical STING signaling pathways are involved in diverse physiological and pathological processes of cellular senescence, autophagy, organ fibrosis, and cardiovascular diseases.

## 3. The dysregulation of the STING pathway in GBM

**Figure 2:**
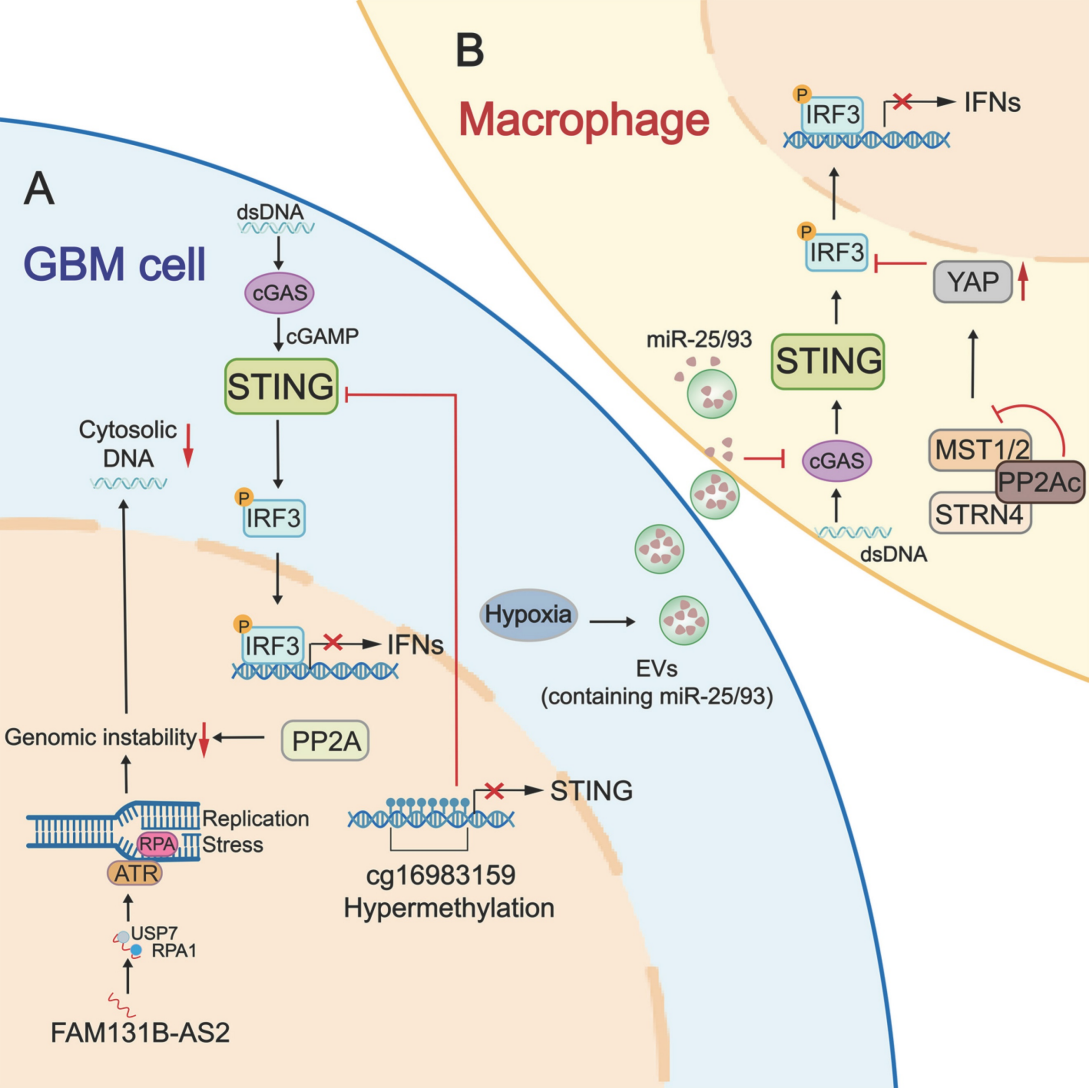
**The dysregulation of the STING pathway in GBM. A:** The dysregulated STING signaling pathway in GBM tumor cells. STING promoter hypermethylation (cg16983159) decreases STING expression in GBM cells, leading to insufficient STING activation. LncRNA FAM131B-AS2 facilitates the repair of replication stress-induced DNA damage by recruiting USP7 to stabilize RPA1 and activate the ATR pathway, thereby diminishing DNA accumulation-mediated STING activation. Similarly, PP2A serves as a negative regulator for STING activation by suppressing cytosolic dsDNA accumulation through enhancing DNA repair and genetic stability. **B:** The dysregulated STING signaling pathway in GBM-associated macrophages. The PP2A/STRN4-Hippo-YAP/TAZ axis can suppress STING-driven type I IFN production in macrophages. Besides, a hypoxic environment promotes the release of EVs from GBM cells and increases miR-25/93 expression in EV cargos. When macrophages take up these EVs, the contained microRNAs hinder the expression of the cGAS protein. Together, these dysregulations blunt STING-mediated immune surveillance. USP7: ubiquitin-specific peptidase 7; RPA1: replication protein A1; ATR: Ataxia Telangiectasia and Rad3-related; PP2A: protein phosphatase 2A; YAP: Yes-associated protein; TAZ: Transcriptional co-activator with PDZ-binding motif; EVs: extracellular vesicles.

The STING signaling pathway serves as the core activator of both innate and adaptive immunity against tumors. However, tumor cells evolve multiple mechanisms to disrupt STING-mediated immune surveillance. Accumulating evidence indicates that the STING signaling pathway is frequently compromised in GBM due to epigenetic silencing, insufficient dsDNA accumulation, PP2A (protein phosphatase 2A)/STRN4 (striatin 4)-Hippo-YAP (Yes-associated protein)/TAZ (Transcriptional co-activator with PDZ-binding motif) axis, and hypoxia-induced inhibition (Figure 2). Clarifying these underlying mechanisms is essential for designing effective, personalized therapies to restore STING-mediated antitumor immunity in GBM.

### 3.1 Epigenetic silencing of STING expression

Single-cell RNA-sequencing and multiplex immunofluorescence assays of GBM patient samples revealed that STING is detected only in immune cells (restricted to myeloid cells) and stromal cells, but is absent in neoplastic and T cells. This absence is attributable not to genetic mutation, as STING mutations are rare in GBM (<1%), but rather to epigenetic repression. Using Illumina methylation arrays, Low* et al.* identified hypermethylation at cg16983159 site of the STING promoter across 64 patient samples and GBM LN229/U138 lines. Cg16983159 hypermethylation correlated with reduced STING expression and conferred STING agonist unresponsiveness. In comparison, a DNA methyltransferase (DNMT) inhibitor decitabine can effectively restore STING expression, re-sensitize cells to STING agonists, activate ISGs (e.g., p-IRF3, IFIT1, pSTAT1, ISG15), and induce innate immune responses in GBM models 23. Unlike stable epigenetic silencing observed in neoplastic cells, the limited STING expression in tumor-infiltrating T cells appears to be a dynamic, regulated state driven by T cell development, dysfunction, or exhaustion 24. However, limited studies have focused on epigenetic silencing of STING expression. In breast cancer, transcription factor FOXM1 can recruit the DNMT1-UHRF1 complex to methylate the STING promoter and thereby suppress its expression 25. Likewise, DNMT1 methylates the STING promoter of T cells from syngeneic mouse tumors and human colorectal cancer 24. These observations implicate roles of FOXM1 and DNMT1 in the epigenetic silencing of STING expression, a mechanism that warrants validation in GBM.

### 3.2 Insufficient dsDNA-induced inhibition of STING activation

In addition to external agonists, intracellular stimuli, particularly cytosolic DNAs, are critical regulators for cGAS-STING-type I IFN axis. In neoplastic cells, high replication stress and genomic instability cause chromosomal abnormalities and DNA damage, generating abundant cytosolic DNA fragments 26, 27. These tumor-derived DNAs directly promote cGAMP synthesis to trigger a potent STING-mediated immune response. In GBM, however, intracellular accumulation of these DNA species is insufficient, restraining STING activation and diminishing tumor immunogenicity. Recent studies have identified PP2A as a key regulator that suppresses cytosolic dsDNA accumulation in GBM cells, limiting STING activation (Figure 2). PP2A is a serine/threonine phosphatase required for DNA damage checkpoint regulation and DNA repair by dephosphorylating DDR (DNA damage response)-related kinases (e.g., ATM, ATR) and γ-H2AX 28, 29. Loss of α-isoform of the catalytic subunit of PP2A (PP2Ac) in glioma cells impairs DNA repair, leading to accumulation of unrepaired dsDNA and cGAMP production. This further enhances cGAS-STING-type I IFN axis and potentiates anti-tumor immunity through promoting DC cross-presentation, CD8⁺ T cell expansion, and reducing immunosuppressive macrophages 30. Similarly, lncRNA FAM131B-AS2, which is frequently upregulated in GBMs with combined +7/-10 alterations, can diminish cytosolic DNA-mediated cGAS activation by promoting DNA repair. Mechanistically, FAM131B-AS2 supports replication stress response in GBM cells by recruiting USP7 to stabilize RPA1 and activate the ATR (Ataxia Telangiectasia and Rad3-related) pathway, thereby protecting single-stranded DNA from breakage. Accordingly, overexpression of FAM131B-AS2 causes diminished phosphorylation of STING, TBK1, and IRF3 in GBM LN229 cells. Silencing FAM131B-AS2 leads to an increase in STING-dependent production of CXCL9, CXCL10, CXCL11, and IFN-β 31. These findings suggest that targeting PP2A, FAM131B-AS2, or the DNA damage repair signaling pathway could be a strategy to recover STING-mediated antitumor immune response in GBM. Besides DNA repair regulators, other DNA-clearing enzymes, such as cytosolic DNA exonuclease TREX and endonuclease DNase II, can degrade cytosolic dsDNA and thereby reduce cGAS-STING signaling pathway activation in tumors like colorectal cancer and melanoma 32-34. Collectively, mechanisms associated with PP2A, FAM131B-AS2, enhanced DNA repair, TREX1, DNase II, and augmented autophagic clearance may constrain STING-mediated type I IFN production and impede effective immune responses in GBM.

### 3.3 Hippo-YAP/TAZ axis negatively regulates STING activation

The PP2A/STRN4-Hippo-YAP/TAZ axis has been shown to suppress STING-mediated type I IFN production in tumor-associated macrophages (TAMs) (Figure 2). Mechanistically, PP2Ac associates with the B subunit STRN4 to bind and dephosphorylates Hippo kinase MST1/2. Inhibited Hippo signaling blocks the phosphorylation, inactivation, and degradation of YAP 35. As a result, the stabilized YAP protein counteracts downstream effectors of the STING signaling pathway, including TBK1 and IRF3 36, 37. This mechanism is particularly relevant in GBM, where YAP is frequently upregulated in GBM-associated macrophages. Overexpressed YAP abolishes the pIRF3 and pSTAT1, impairs type I IFN production, and reduces responsiveness to STING agonists. By contrast, deficiency of PP2A, STRN4, or YAP in TAMs can enhance type I IFN signature (IFNβ, CXCL10, CXCL9, and ISG15), remodeling the immune landscape with increased CD8^+^/CD4^+^ T cell infiltration in orthotopic glioma and decreased tumor burden in subcutaneous glioma 35. These observations indicate the significance of PP2A/STRN4-Hippo-YAP/TAZ signaling in negatively regulating the STING signaling pathway in GBM. Targeting this pathway can enhance the sensitivity to STING agonists and prime anti-GBM immunity.

### 3.4 Hypoxia-mediated suppression of STING activation

Hypoxia is a hallmark of cancers and contributes to the dysregulated activity of the STING signaling pathway. In glioma, hypoxia induces the impairment of STING activation in immune cells through a mechanism associated with tumor-derived extracellular vesicles (EVs) (Figure 2). Hypoxia promotes EV release from GBM cells and increases miR-25/93 expression in both cells and EV cargos. When macrophages take up these EVs, the contained microRNAs hinder the expression of the cGAS protein. Thus, downstream STING-TBK1-IRF3 signaling is attenuated in macrophages, leading to reduced production of IFN-α, IFN-β, CXCL9, CXCL10, and IL-12, and impairing T cell recruitment and activation 38.

In summary, the STING signaling pathway in GBM and immune cells is often impaired by various regulatory mechanisms, creating a significant challenge to antitumor immunity. Personalized therapies by targeting specific mechanisms offer a promising strategy to restore STING activity and enable effective tumor control.

## 4. The roles of the STING signaling pathway in overcoming immunosuppressive GBM

**Figure 3:**
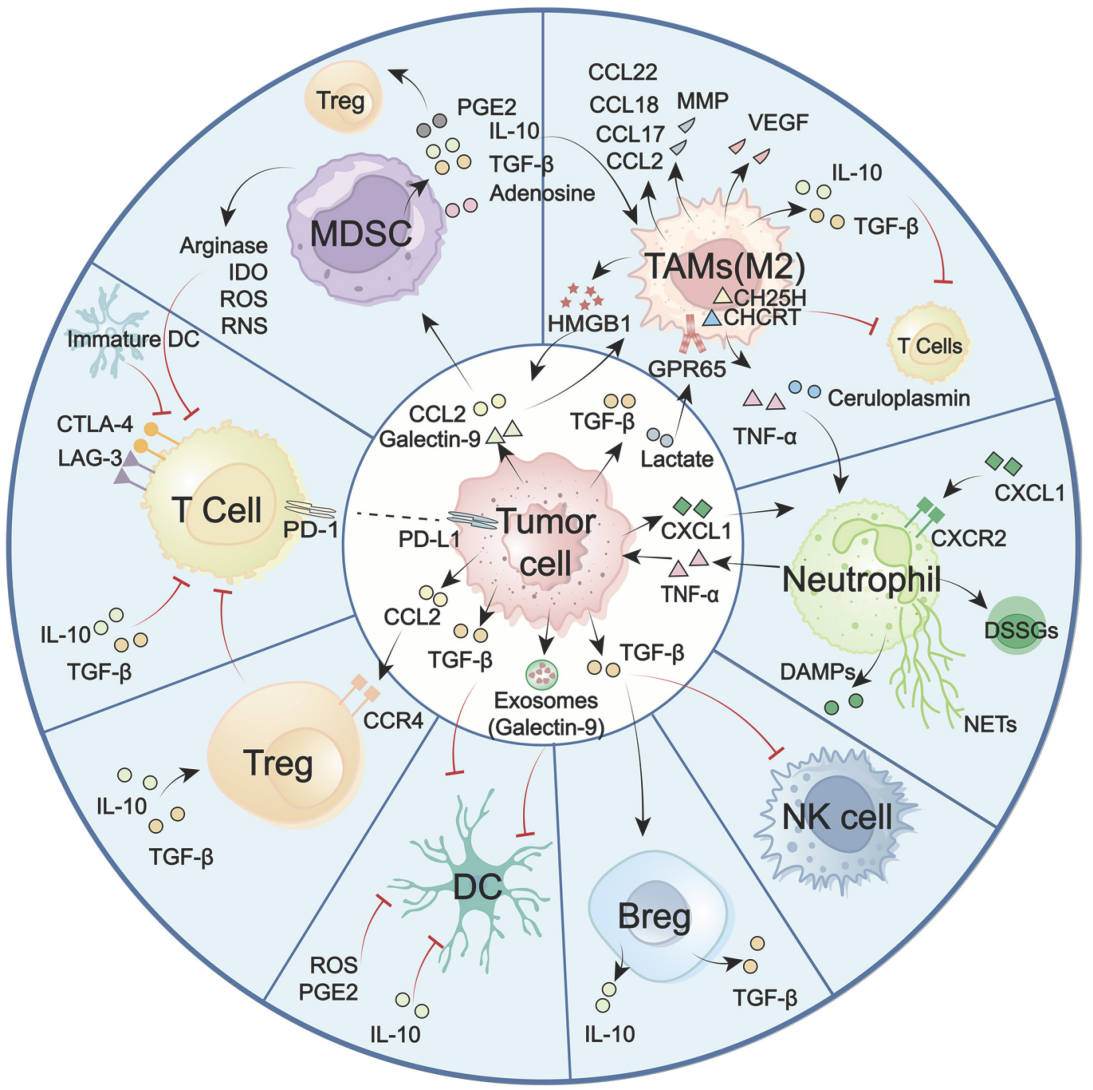
**The immune cells drive GBM “cold” environment.** GBM TME is predominant with immunosuppressive myeloid cells, notably TAMs and MDSCs. In contrast, cytotoxic T cells, NK cells, and DCs are scarce or functionally constrained. These cellular constituents assemble an integrated immunosuppressive network to support tumor malignancy. Typically, neoplastic cells and immunosuppressive cells (e.g., M2-like TAMs, MDSCs, Tregs, Bregs) secrete numerous cytokines (e.g., TGF-β, TNF-α, IL-10), chemokines (e.g., CCL2, CCL17, CCL18, CCL22), metabolic enzymes (e.g., Arginase, IDO), matrix-remodeling enzymes (MMPs), reactive oxygen species (ROS), and other immune-inhibitory mediators. These factors collectively drive M2 macrophage polarization, upregulate immune checkpoint molecules, impair recruitment and function of cytotoxic T and NK cells, and disrupt antigen presentation. This intricate immunosuppressive crosstalk fosters a profoundly suppressive TME and compromises anti-tumor immunity. TME: tumor microenvironment; TAMs: tumor-associated macrophages; MDSCs: Myeloid-derived suppressor cells; NK cells: natural killer cells; DC(s): dendritic cell(s); Tregs: regulatory T cells; Bregs: regulatory B cells; DSSGs: disease-specific suppressive granulocytes; NETs: neutrophil extracellular traps.

**Figure 4:**
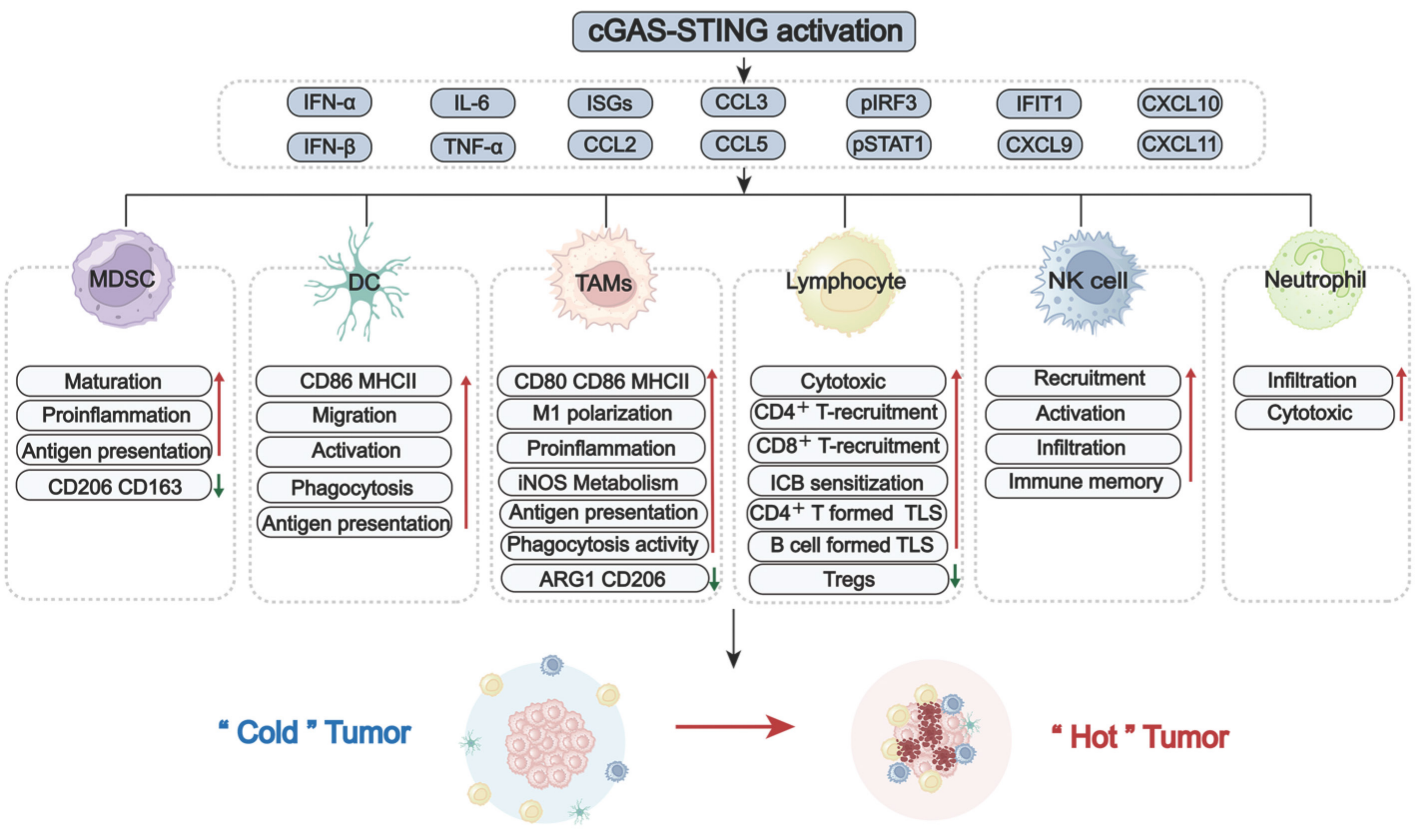
** STING signaling pathway-driven reprogramming of the GBM immune landscape.** Activation of the cGAS-STING signaling pathway potently stimulates innate interferon responses and pro-inflammatory signaling, playing a pivotal role in remodeling the immunosuppressive landscape of GBM. Upon activation, the STING signaling pathway induces type I IFNs, cytokines (e.g., IL-6, TNF-α), interferon-stimulated genes (ISGs), and chemokines (e.g., CXCL9/10/11, CCL2/3/4). This secretory milieu promotes recruitment, differentiation, maturation, and priming of various immune cells (e.g., MDSCs, DCs, TAMs, NK cells, neutrophils). STING activation can shift TAMS from a suppressive state toward an inflammatory and phagocytic phenotype. Furthermore, it facilitates the infiltration and enhances the effector function of CD8⁺ T cells. TAMs: tumor-associated macrophages; MDSCs: myeloid-derived suppressor cells; NK cells: natural killer cells; DC(s): dendritic cell(s); Tregs: regulatory T cells; ICB: Immune Checkpoint Blockade; TLS: tertiary lymphoid structures.

GBM faces significant challenges, including high tumor-intrinsic heterogeneity, hypoxia, and the blood-brain barrier (BBB). These factors collectively contribute to a unique and immunosuppressive landscape, marked by the dominance of immunosuppressive cells, including TAMs, MDSCs, and lymphoid-derived regulatory T cells (Tregs). In contrast, key antitumor effector populations, including cytotoxic CD8⁺ T cells, NK cells, and professional antigen-presenting DCs, are often scarce or functionally impaired 39, 40. The imbalance between these suppressive and effector immune cell subsets, along with their dynamic crosstalk with tumors and TME, represents a major obstacle for effective immune surveillance (Figure 3). Overcoming this challenge requires strategies to reprogram the immune landscape, a process in which the STING signaling pathway has emerged as a pivotal regulator. Increasing evidence highlights the critical role of the STING signaling pathway in counteracting immunosuppressive TME by altering immune cell recruitment, differentiation, polarization, and antigen presentation. To systematically elucidate STING-mediated immunomodulatory mechanisms in GBM, the following overview delineates how major immune cell subsets establish immunosuppressive TME (Figure 3). Also, the role of the STING signaling pathway in reprogramming these cells to restore antitumor immunity and improve therapeutic responses is summarized (Figure 4).

### 4.1 Myeloid-lineage cells

In the GBM TME, myeloid-lineage cells, particularly TAMs and MDSCs, comprise the dominant immune compartment. Pro-tumoring TAMs and MDSCs contribute to profound immunosuppression by impairing T cell function and suppressing NK cell and DC activity. Targeting these myeloid-lineage cells or modulating their activity represents a promising strategy for alleviating immunosuppression and restoring effective antitumor immunity.

#### TAMs

TAMs, comprising both brain-resident microglia and bone marrow-derived monocytes, account for up to 50% of total tumor mass 41, 42 (Figure 3). Driven by stimulation from neoplastic cells and the environment, TAMs primarily polarize into classically activated macrophages (M1-like) and alternatively activated macrophages (M2-like). M1-like TAMs can directly kill tumor cells through secreting inflammatory factors, including ROS, TNF-α, IL-6/23, and CXCL9/10/16. They can also boost adaptive antitumor immunity by upregulating antigen-presentation molecules such as MHC II and CD80/CD86 43. M2-polarized TAMs perform protumor functions. By releasing cytokines (e.g., IL-10, TGF-β, CCL2/7/18/22), growth factors (e.g., VEGF), and enzymes (MMPs), M2-TAMs promote angiogenesis, impair T cell function, and induce therapy resistance. Thus, the predominance of this M2 phenotype in the TME is a major contributor to immune evasion 44, 45.

Regarding the protumor role of M2-TAMs, strategies to reprogram them, typically by encouraging M1 polarization, hold therapeutic promise. This reprogramming can be facilitated by the STING pathway effector molecules IFN-γ and TNF-α 36, 39, 44, 45, making STING activation a rational and compelling therapeutic strategy. *In vitro* experiments have demonstrated that directly activating STING signaling pathway in TAMs induces expression of pro-inflammatory genes (e.g., type I IFNs, CCL5, and CXCL10), upregulates CD80/CD86 and MHC II, while reducing immunosuppressive factors arginase 1 (ARG1) and CD206 35, 46-48. In addition, STING activation within neoplastic cells can exert a paracrine effect that reprograms adjacent TAMs toward an M1-like phenotype, resulting in a 9-fold increase in the M1/M2 ratio 49. Supporting these observations, STING agonists have been shown to reduce the proportion of M2-type macrophages in an orthotopic glioma mouse model 47, 48. Beyond repolarization, STING activation also shifts TAM metabolism from polyamine synthesis to the iNOS/NO pathway, further supporting its pro-inflammatory function 48. These results collectively establish the role of STING signaling pathway in reprogramming TAMs by promoting their repolarization or modifying their immunosuppressive properties in GBM (Figure 4).

#### MDSCs

MDSCs are a heterogeneous population originating from common myeloid progenitors in the bone marrow. Recruited by tumor-secreted cytokines and chemokines (CCL2 and Galectin-9), MDSCs infiltrated into GBM TME 50. Different from the MDSCs-differentiated mature macrophages, DCs, or granulocytes, MDSCs remain in an immature and protumor state 51 (Figure 3). Through the release of immunosuppressive cytokines (TGF-β and IL-10), amino acid-depleting enzymes (arginase and IDO), and ROS/nitrogen species, MDSCs repolarize macrophages, expand Tregs, and abrogate T cell proliferation, contributing to a broadly immunosuppressive landscape 52-57. Interestingly, MDSCs exhibit heterogeneity among gliomas and account for the poorer prognosis. Metabolically active early progenitor MDSCs are notably enriched in IDH-wildtype GBM, while CXCR4⁺ monocytic-MDSCs (M-MDSCs) frequently accumulate post radiotherapy. The heterogeneity of MDSCs also depends on gender. M-MDSCs accumulate in male tumors, whereas granulocytic MDSCs predominate in female peripheral blood 55, 57-59.

Accumulating evidence indicates that the STING-type I IFN axis can promote MDSC differentiation toward a more mature, immunostimulatory state (Figure 4). In GBM preclinical models, as measured by *ex vivo* flow cytometry, STING agonists can increase brain influx of CD11b⁺Ly6C⁺ monocytic MDSCs and reprogram them toward a pro-inflammatory, antigen-presenting phenotype with decreased immunosuppressive markers CD206/CD163 19, 48. In line with this, research in melanoma and colon cancer found that STING activation reduced MDSC abundance, suppressed their immunosuppressive activity, and thereby inhibited tumor metastasis 60. However, studies involving GL261 subcutaneous tumors indicate that the therapeutic benefit of STING agonists can be abrogated by the expansion of granulocytic MDSCs 61. This observation suggests a more effective approach that combines agonists with MDSC targeting.

#### DCs

DCs are professional APCs that initiate and regulate both innate and adaptive immune responses 62. However, in response to tumor-derived exosomes or myeloid cell-generated immunosuppressive mediators (IL-10, TGF-β, ROS, and PGE₂), DCs experience a functional shift in GBM 51, 63, 64. They exhibit heterogeneity and are classified into four subsets: cDC1, which cross-present tumor antigens to activate CD8⁺ T cells; cDC2, which prime CD4⁺ T cells; pDCs, which produce type I IFNs with context-dependent effects; and moDCs, which display an immunosuppressive phenotype 65, 66. Notably, most DCs display an immature phenotype, marked by reduced antigen presentation and ineffective T cell priming (Figure 3), making them attractive candidates for novel immunotherapeutic targeting.

The STING signaling pathway is critical for DC activation, as type I IFNs and proinflammatory cytokines are required for antigen presentation (Figure 4). In response to IFN-β, the migration, phagocytosis, and maturation of DCs were promoted in both U251 and T98G glioma cells 67. Animal studies utilizing the orthotopic GL261 model also found increased DC maturation in draining lymph nodes following cGAS-STING activation, enhancing T-cell responses 68. Besides, the administration of a STING agonist in the GBM QPP8 model increased CD86 expression on DCs and expanded antigen-cross-presenting cDC1s in both the TME and cervical draining lymph nodes. These insights underscore the potential of STING-based strategies to reprogram DCs in GBM 19.

#### Neutrophils

Neutrophil accumulation correlates with tumor progression and poor prognosis in GBM 69. Upon recruitment to the brain, neutrophils undergo functional reprogramming in response to TNF-α and ceruloplasmin driven by tumor-associated myeloid cells (TAMCs) 70. Hence, tumor-associated neutrophils (TANs) exhibit different characteristics from neutrophils in peripheral blood. TANs display prolonged survival, pro-angiogenic activity, and immunosuppressive properties (Figure 3). Functionally, TANs modulate tumor metabolism and drive mesenchymal transition via the secretion of TNF-α and acid ceramidase ASAH1. TANs also support an immunosuppressive TME by releasing danger-associated molecular patterns (DAMPs) 69, 71 or neutrophil extracellular traps (NETs) 72, 73. Besides the protumor TANs, recent work has identified a distinct antitumor TAN subset derived from skull marrow precursors. These TANs are dendritic-like, capable of enhancing MHC II-dependent T-cell activation 74. These results highlight the plasticity and heterogeneity of neutrophils in GBM. Repolarizing the protumor into an antitumor phenotype or recruiting antitumor neutrophils holds promise for therapeutic applications.

The STING signaling pathway is crucial for neutrophil recruitment and function (Figure 4). Across multiple tumor models, including breast cancer, melanoma, and Lewis lung carcinoma, intratumoral STING activation with cGAMP promoted Ly6G^+^ neutrophil recruitment via activating the NF-κB-CXCL1/2-CXCR2 axis. Additionally, IFN-β1-stimulated neutrophils displayed enhanced tumor cell killing, suggesting the requirement of the STING-IFN axis for neutrophil-mediated cytotoxicity 75, 76. Consistent with these findings, STING stimulation in GBM models elicited robust neutrophil responses. In the mice bearing GL261/CT-2A tumors, agonist ADU-S100 triggered substantial infiltration of cytotoxic neutrophils, remodeled the TME, and significantly prolonged survival 5.

### 4.2 NK Cells

NK cells are innate cytotoxic lymphocytes capable of eliminating malignant cells independent of prior sensitization. In GBM, NK cells are scarce and exhibit markedly impaired function (Figure 3). Influenced by soluble and contact-dependent immunosuppressive cues, NK cells often display an immature CD56⁺ CD16⁻ phenotype or express high levels of inhibitory receptors like PD-1, LAG-3, and TIGIT, contributing to reduced cytotoxic function 77-79. Therefore, therapeutic strategies aimed at restoring NK activity through utilizing engineered NK cells 80, 81, NK cell-based oncolytic viruses 82, or NK cell programming have achieved significance in GBM preclinical models 83, 84.

The STING signaling pathway is another approach to restore NK cell-mediated anti-tumor responses by producing IFN-inducible chemokines (CXCL9/10/11). These chemokines interact with CXCR3 on NK cells, stimulating their recruitment and activation (as shown in Figure 4). Consistently, in multiple murine intracranial glioma models, treatment with STING agonists reshaped the brain TME with massive infiltration of NK cells, resulting in long-term survival and immune memory 5, 19. Moreover, it has been reported that STING agonists, when combined with NK-based therapies, can synergistically enhance anti-tumor effects in GBM 5.

### 4.3 Lymphoid cells

#### CD8⁺ and CD4⁺ T cells

Most lymphocytes in the brain are recruited from the peripheral circulation. Thus, recent evidence suggests cranial bone marrow adjacent to GBM is a reservoir for CD8⁺ T cells 85. Although there is potential for T cell involvement, their infiltration into brain tumors remains limited due to several obstacles like BBB, dense extracellular matrix, inhibitory chemokines, immune checkpoints, and glucocorticoid-mediated T cell sequestration. Hence, CD4⁺ and CD8⁺ T cells are notably scarce and insufficient to exert tumor removal 86 (Figure 3). Additionally, the scarce CD8⁺ T cells are often exhausted with elevated levels of PD-1, CTLA-4, and LAG-3 expression 87, 88. This exhaustion is associated with the JAK/STAT pathway and immunosuppressive cytokines driven by myeloid cells 51, 87, 89, 90. CD4⁺ helper T cells also show impairments in proliferation and function, which further contribute to CD8⁺ T cell exhaustion and reduce PD-1 blockade efficacy 87, 91, 92. This substantial dysfunction in both CD8⁺ and CD4⁺ T cells constitutes a significant barrier to the establishment of effective adaptive immunity.

The STING signaling pathway serves as a crucial link between innate and adaptive immune responses (Figure 4). As previously mentioned, the STING-IFN axis greatly enhanced the antigen-presenting capacity of myeloid cells, triggering robust T cell infiltration and activation 48. Meanwhile, the recruitment and activation of T cells rely on IFN and chemokine stimulation. Increased IFN-β and CXCL9/10/11 in GBM cells, driven by phosphorylation of STING and IRF3, directly promoted the infiltration and proliferation of CD8^+^ T cells and ultimately improved the response to PD-1 inhibitors 31. Similarly, STING-TBK1 activation in GL261 and NPA glioma cells via Chek2 inhibition enhanced the cytotoxicity of co-cultured CD8⁺ T cells 93. Cheng *et al.* further confirmed this mechanism utilizing GBM-bearing mice. They found increased CD3^+^CD4^+^ and CD3^+^CD8^+^ T cell infiltration in tumor TME, with 3.8- and 3.5-fold higher compared to controls, following cGAS-STING-IFN-β/CXCL10 axis stimulation 49. Thus, accumulating evidence has illustrated the critical role of STING activation in priming T cells in GBM, offering new hope for overcoming immunosuppression.

#### Tregs

Tregs only represent a minor fraction of GBM-infiltrating lymphocytes. However, they have a potent immunosuppressive function that markedly impairs T cell activity 94, 95 (Figure 3). Several Treg clusters have been identified. For instance, radiotherapy-induced infiltration of CD103⁺ Tregs in GBM can suppress CD8⁺ T cell function and dampen the response to immune checkpoint blockade 39. CD25⁺ Tregs can induce TME reprogramming by suppressing CD8⁺ T cell activation and myeloid phagocytosis 96. Due to their immunosuppressive function, strategies such as depleting Tregs or inhibiting their metabolism present promise for resensitizing GBM to immunotherapy and chemotherapy 97, 98.

The role of the STING signaling pathway in regulating Tregs has been reported in multiple tumor models. In GBM preclinical studies, STING activation significantly reduced the proportion of Tregs in the TME, from 37.12% in controls to 18.35% in treated mice 49.

#### B cells

B cells are present at low frequencies in the GBM TME and have both anti-tumor and pro-tumor functions (Figure 3). On the anti-tumor aspect, B cells form tertiary lymphoid structure (TLS)-like aggregates near T cells, supporting local antigen presentation and promoting T-cell activation. B cell-based vaccine strategies, such as BVax (CD40- and IFNγ-stimulated 4-1BBL⁺ B cells), have shown promise in inhibiting tumor cell migration and invasion 99. However, when B cells differentiate into immunosuppressive regulatory B cells (Bregs), they acquire a protumor phenotype that promotes tumor progression by secreting IL-10 and TGF-β 100, 101. STING has also been reported to expand B cells and amplify B cell-formed TLS in GBM models by driving IFN-α, IFN-β, and TNF-α release 47.

As summarized above, the regulatory functions of canonical STING-TBK1-IRF3-type I IFN axis in immune cells are well-established. In contrast, the biological significance of STING non-canonical branches in the GBM TME remains poorly defined. Evidence from other tumor types suggests that non-canonical pathways can critically modulate the TME through crosstalk with the canonical axis. One key example is the non-canonical NF-κB (RelB/p52) pathway. Once activated, it engages a negative feedback that restrains canonical STING-type I IFN production in APCs, thereby dampening T-cell priming. Genetic disruption of RelB/p52 in tumor murine models enhanced STING-dependent antitumor immunity and improved radiosensitization 16, 102. PERK activation, another non-canonical STING signaling, can maintain nuclear factor erythroid 2-related factor 2 (NRF2)-mediated antioxidant capacity and mitochondrial respiratory homeostasis in MDSCs. This regulatory mechanism limited cytosolic mitochondrial DNA accumulation, which constrained STING-mediated MDSC transformation and immunity 103. These findings position non-canonical STING pathways as potential immunosuppressive regulators within the TME by attenuating canonical STING activity.

In summary, GBM TME has a dysfunctional and immunologically “cold” nature. Its core characteristic is the dominance of suppressive myeloid-lineage cells, particularly TAMs and MDSCs. These cells create a suppressive niche that drives cytotoxic T cell exhaustion and infiltration failure, while also impairing other effector populations such as NK cells and DCs (Figure 3). This extensive, multifaceted suppression underlies GBM immune evasion and progression. Increasing evidence has illustrated that STING activity can significantly impact immune cell recruitment, differentiation, activation, and cytotoxicity, thereby priming anti-GBM immunity (Figure 4). Hence, STING has emerged as a compelling therapeutic target and considerable effort is being made to develop its agonists for GBM treatment.

## 5. STING agonists in GBM

**Figure 5:**
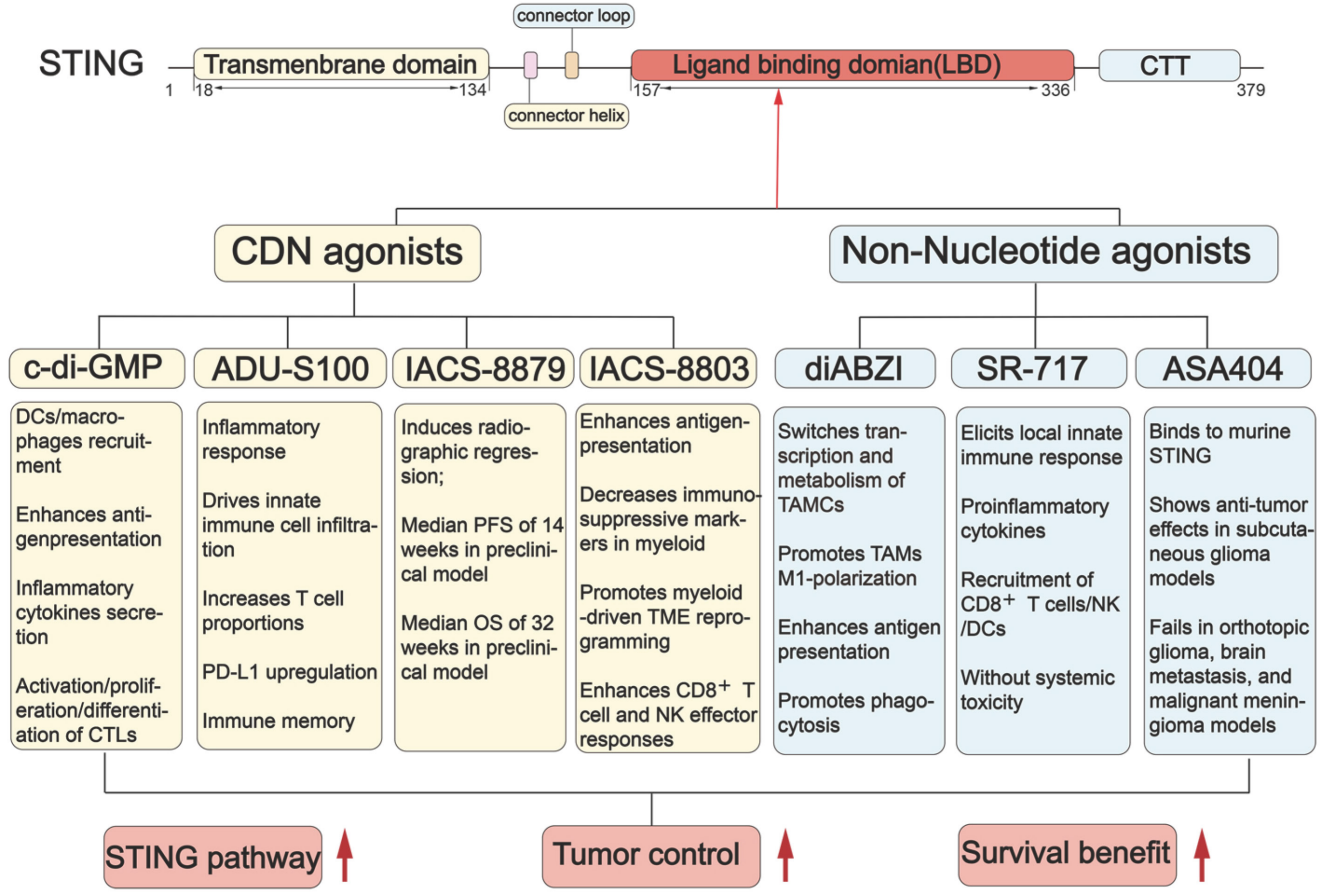
**The STING structure and the mechanism of STING agonists in GBM treatment.** STING has a defined ligand-binding domain (LBD) in its C-terminal cytosolic head, which can be recognized by STING agonists. When binding with LBD, STING agonists induce conformational changes in STING, shifting it into an active state. Both cyclic dinucleotide (CDN) agonists (e.g., c-di-GMP, ADU-S100, IACS-8779, and IACS-8803) and non-nucleotide small-molecule agonists (e.g., diABZI, SR-717, ASA404) activate the STING pathway to trigger a proinflammatory response. This activation promotes immune cell remodeling and initiates anti-tumor immunity. DC(s): dendritic cell(s); TAMs: tumor-associated macrophages; NK cells: natural killer cells; CTLs: cytotoxic T lymphocytes; TAMCs: tumor-associated myeloid Cells; PFS: Progression-Free Survival; OS: Overall Survival.

**Figure 6:**
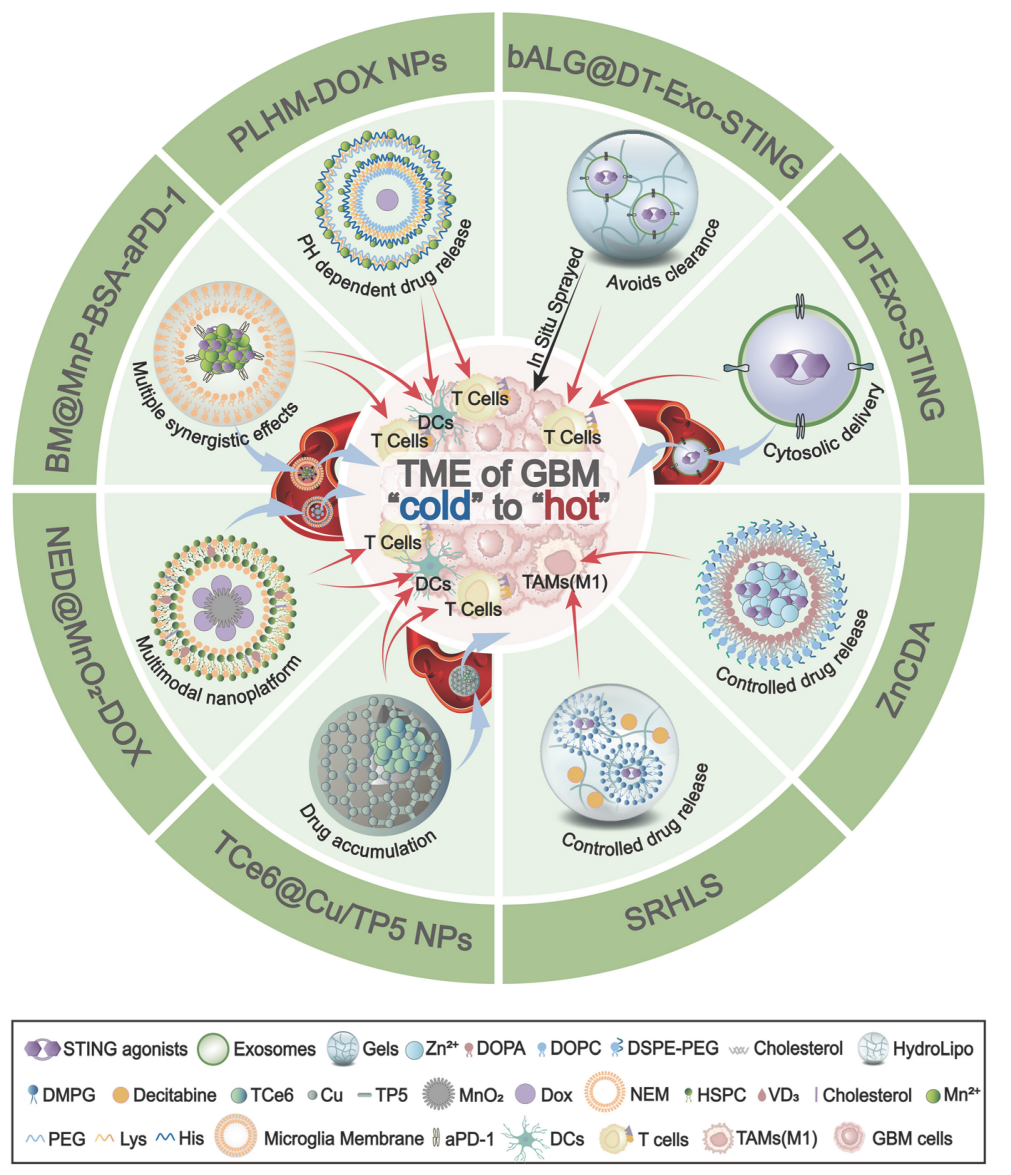
** The innovative delivery platforms in GBM.** Schematic overview of nanoplatforms that deliver STING agonists to remodel the immunologically “cold” TME of GBM into a “hot” state. DT-Exo-STING: An immunotherapeutic system based on chimeric exosomes derived from dendritic cell (DC)-tumour hybrid cells. It restores the activity of STING agonists, enhances antigen presentation by DCs, and promotes robust T-cell priming. bALG@DT-Exo-STING: An artificial lymph-node-like depot formed by *in situ* spraying of an alginate gel cross-linked with DT-Exo-STING. The bioorthogonal reaction between azide-modified exosomes and alkyne-modified alginate prolongs local retention, avoids rapid clearance, and drives potent T-cell activation. ZnCDA: A nanoscale coordination polymer encapsulating bacterial cyclic dimeric adenosine monophosphate (CDA). It enhances intratumoral accumulation, selectively targets tumour-associated macrophages (TAMs), and boosts STING-dependent antigen presentation and T-cell responses. SRHLS: A sequential release hydroLipo system integrating hydrogels and nanoparticles to achieve spatiotemporally controlled delivery. SRHLS releases decitabine followed by STING agonists to potentiate antitumour immunity. PLHM-DOX NPs: Telodendrimer/Mn²⁺-based doxorubicin (DOX) nanoparticles. By combining Mn²-mediated potentiation of the STING pathway with cytosolic DNA accumulation, PLHM-DOX NPs activate innate immunity, promote DC maturation, and drive infiltration of CD8⁺ cytotoxic T cells into GBM. TCe6@Cu/TP5 NPs: A brain-targeted nanoassembly formed by self-assembly of triphenylphosphonium-conjugated Chlorin e6 (TPP-Ce6, TCe6), copper ions, and thymopentin (TP5). TCe6@Cu/TP5 NPs trigger mitochondrial photodynamic damage, activate the AMP-activated protein kinase (AMPK) pathway to promote PD-L1 degradation, and engage the cGAS-STING pathway to enhance antitumour immunity. BM@MnP-BSA-aPD-1: BV2 microglia-membrane-coated manganese porphyrin-BSA nanoparticles conjugated with anti-PD-1 antibody. BM@MnP-BSA-aPD-1 crosses the BBB, targets the GBM TME, activates the cGAS-STING pathway to promote DC maturation, activate CD8⁺ T cells and NK cells, and sensitizes tumours to aPD-1 therapy. NED@MnO₂-DOX: A multimodal platform in which vitamin D₃-inserted lipid hybrid neutrophil membrane (NED) cloaks MnO₂-based DOX-loaded nanoparticles. NED@MnO₂-DOX exploits neutrophil-like tropism to reach GBM, decomposes to generate Mn²⁺, and activates the cGAS-STING pathway. BBB: blood-brain barrier; CDA: cyclic dimeric adenosine monophosphate; Ce6: Chlorin e6; DOX: doxorubicin; NPs: nanoparticles; TP5: thymopentin; BM: BV2 microglia membrane; SRHLS: sequential release HydroLipo system; DCs: dendritic cells; TAMs: tumour-associated macrophages; TME: tumour microenvironment.

Since the STING signaling pathway is suppressed in GBM, STING agonists have emerged as a promising strategy for treating GBM. STING agonists are a class of compounds that bind to STING and effectively activate the STING signaling pathway (Figure 5) 104. The development of STING agonists progresses through three main phases. It starts with nucleotide-based agonists, moves to non-nucleotide small molecules, and is currently towards innovative platform-based agonists. Nucleotide-based agonists are the earliest efforts, designed to mimic endogenous STING ligands. This class includes foundational cyclic dinucleotides (CDNs) like c-di-GMP and more advanced analogues, such as ADU-S100, IACS-8779, and 8803. Although these molecules have been validated as effective in treating preclinical GBM models, their clinical translation has been hindered due to poor stability and low permeability across the BBB 105. To address these limitations, increasing efforts are made to develop non-nucleotide small-molecule agonists. Unlike the charged and hydrophilic CDNs, these compounds are improved in drug properties, including smaller size, more lipophilicity, and more stability 106. diABZI, SR717, and ASA404 are prominent examples in this class well investigated for glioma. Currently, to further enhance the efficiency and safety of STING agonists, innovative therapeutic platforms, such as nanoparticle delivery systems, have been developed to deliver these agents. Here, we provide an overview of the landscape of STING agonists for GBM treatment and discuss their advancements with novel delivery systems, highlighting the potential for these agonists to improve clinical outcomes (Figure 5-6, Table 1-2).

### 5.1 CDN agonists

#### Cyclic-di-GMP

Cyclic-di-GMP (cdGMP), also known as c-di-GMP, is a type of cyclic dinucleotide derived from bacterial sources. CdGMP can activate the STING signaling pathway to stimulate the innate immune response. In GBM, cdGMP successfully induces potent anti-tumor immunity and provides early proof for therapeutic approaches utilizing STING agonists 107 (Figure 5, Table 1). In the GBM GL261 orthotopic mice model, cdGMP promotes the release of IFN-β from APCs, recruits DCs and macrophages to the TME, and subsequently enhances CD8^+^ T cell activity. However, cdGMP is susceptible to endosomal degradation, limiting its cytosolic delivery and effectiveness 108, 109. To address this, nanoparticle systems are developed to enhance cdGMP-mediated immune activation 108. For example, a cationic poly (ester amide)-based nanoparticle enhanced the cytosolic delivery of cdGMP, promoted DC maturation, antigen presentation, and cytokine secretion. Activated DCs can effectively trigger activation, proliferation, and differentiation of antigen-specific cytotoxic T lymphocytes, driving robust immune protection against tumors 110. These strategies present a promising approach for enhancing the delivery and efficacy of cdGMP in treating GBM.

#### ADU-S100

ADU-S100, also known as MIW815 or ML-RR-S2-CDA, is a kind of bisphosphothioate analog of cyclic di-AMP with the CDN construct. This structural improvement confers greater resistance to enzymatic hydrolysis and a higher binding affinity for STING. Consequently, ADU-S100 enhances the capacity to induce a potent inflammatory response and anti-tumor immunity. The therapeutic efficacy of ADU-S100 has been widely investigated in GBM preclinical models (Figure 5 and Table 1). In murine GL261/CT-2A models, ADU-S100 induced a concentration-dependent infiltration of innate immune cells. Massive inflammatory macrophages, neutrophils, and NK cells were recruited into the tumor-bearing brain, establishing significant immune memory and leading to prolonged survival 5. However, ADU-S100 failed to activate its downstream effectors in GBM cells overexpressing lncRNA FAM131B-AS2, as lncRNA FAM131B-AS2 can negatively regulate the phosphorylation of STING, TBK1, and IRF3 31. This finding suggests that GBM cell responses to exogenous agonists can be abrogated by endogenous STING inhibition. However, there is a notable absence of clinical trials evaluating ADU-S100 in glioma treatment, indicating a need for further exploration.

#### IACS-8779

IACS-8779 is a potent small-molecule cyclic dinucleotide STING agonist discovered in 2019 111. IACS-8779 activates the STING signaling pathway to trigger a strong innate antitumor immune response, particularly in immunologically “cold” tumors such as GBM (Figure 5 and Table 1). In a Phase I trial with dogs with spontaneously arising GBM, intratumoral administration of IACS-8779 (5-20 μg) significantly increased antitumor immunity. IACS-8779 induced a dose-dependent tumor reduction, with a median progression-free survival of 14 weeks and a median overall survival of 32 weeks 112. These findings provide a strong foundational basis for the future clinical translation of IACS-8779 for GBM treatment.

#### IACS-8803

IACS-8803 is a synthetic, high-potency CDN STING agonist that can activate human STING and drive pronounced effects in myeloid cells 113. In orthotopic glioma QPP models, IACS-8803 significantly elicited a survival benefit by increasing CD45^+^ immune cell infiltration, converting immunosuppressive microglia into a proinflammatory phenotype, and enhancing responses of CD8^+^ T cells and NK cells 19. Importantly, as the efficacy of IACS-8803 is primarily mediated through the stimulation of myeloid cells, its effect is usually retained even when STING is silenced in tumor cells 19. Thus, IACS-8803 is a promising therapeutic candidate for remodeling myeloid cells for GBM immunotherapy.

### 5.2 Non-nucleotide small-molecule agonists

#### Diaminobenzimidazole (diABZI)

As a pioneering non-nucleotide agonist, diABZI represents a significant advance over first-generation CDNs. diABZI is a potent, systemically active STING agonist that induces robust anti-tumor immunity following intravenous administration 114. *In vivo* treatment with diABZI-loaded nanoparticles remodels the transcriptomic and metabolic features of TAMCs. It promotes the secretion of pro-inflammatory cytokines, including type I IFNs, CCL5, and CXCL10, and converts immunosuppressive TAMCs into antitumor phenotypes. Then these TAMCs recruit and activate T cells in brain tumors 48. In a pediatric high-grade glioma (pHGG) mouse model, the combination of diABZI with radiotherapy and DNA repair inhibitors promotes the formation of antitumor immune memory and improves efficacy 115. However, evidence supporting the monotherapy of diABZI in GBM models remains limited (Figure 5 and Table 1) and needs further investigation.

#### SR-717

SR-717 is a potent non-nucleotide STING agonist and represents a significant advance following diABZI. SR-717 acts as a cGAMP mimetic, which can promote STING's closed conformation and thereby activate the downstream signaling. SR-717 encourages the activation of CD8^+^ T cells, NK cells, and DCs. By facilitating the antigen cross-priming, SR-717 exerts antitumor activity across multiple cancers 116. In GBM studies, SR-717 treatment markedly stimulated the STING signaling pathway, increasing the mRNA levels of IFN-β1, CXCL10, CXCL9, and TNF-α in the human monocyte cell line. SR-717 reduced tumor growth by 55%, improved physical status by slowing the decline in body weight, and extended the median survival of the GL261 tumor-bearing mice compared with the control group 7 (Figure 5 and Table 1). Interestingly, SR-717 can upregulate clinically relevant immunomodulatory targets such as PD-L1 in a STING-dependent manner 116, providing a strong rationale for combining this active agent with PD-1/PD-L1 checkpoint inhibitors for GBM treatment.

#### ASA404

ASA404, also known as 5,6-dimethylxanthenone-4-acetic acid (DMXAA) or Vadimezan, is a potent agonist of murine STING 117. Multiple studies have investigated the potential of ASA404 for treating tumors (Figure 5, Table 1). In glioma, ASA404 produced apparent anti-tumor effects in a subcutaneous U87 model by inducing STING-dependent macrophage recruitment. However, ASA404 did not show any therapeutic benefit in the orthotopic glioma, brain metastasis, and malignant meningioma mouse models 118. These results indicate its low brain penetration and insufficient intracranial drug concentrations. Additionally, ASA404 failed to inhibit the growth of U-87 and LN-229 GBM cells, due to its lower affinity for human STING proteins 118. These pharmacokinetic and species-specific limitations hinder its clinical translation for human application.

### 5.3 Innovative delivery platforms

While non-nucleotide agonists improve pharmacological properties over early CDN agonists, they still face several challenges. Poor brain penetration, systemic toxicity, instability, short half-lives, and poor oral bioavailability hinder their clinical translation. Consequently, recent research has been increasingly focused on developing advanced delivery platforms to improve these limitations (Figure 6 and Table 2). Biomimetic carriers, such as chimeric exosomes derived from DC-tumor hybrids, help enhance the BBB crossing 119. Lymph node-mimicking exosome gels in the resection cavity can locally activate and sustain tumour-infiltrating T cells 120. Moreover, nanoscale coordination polymers are utilized to enhance agonist stability and improve the precise delivery to TAM 46. Nanoplatforms, such as incorporating systems for sequential drug release 121 or integrating metal ions like Mn²⁺ and Copper to augment cGAS-STING activation 68, 122, are employed to harness multi-modal synergy and boost therapeutic efficacy. More importantly, cell membrane-based biomimetic delivery systems have shown promise in improving drug delivery and enhancing STING activation in glioma. These biomimetic nanocarriers utilize natural microglial or neutrophil membranes to amplify immune responses and reverse “cold” gliomas to “hot” tumors. This is because the natural microglial or neutrophil membranes possess "tumor chemotaxis" and "BBB penetration" properties for loading STING activators or agonists 123, 124. Collectively, these innovative delivery approaches enhance the effectiveness of STING agonists or activators in activating the STING signaling pathway, thereby promoting DC maturation, increasing CD8^+^ T cell cytotoxicity, and converting immunologically “cold” GBM into “hot” tumors (Figures 6 and Table 2).

## 6. STING agonists combined with other therapies in treating GBM

**Figure 7:**
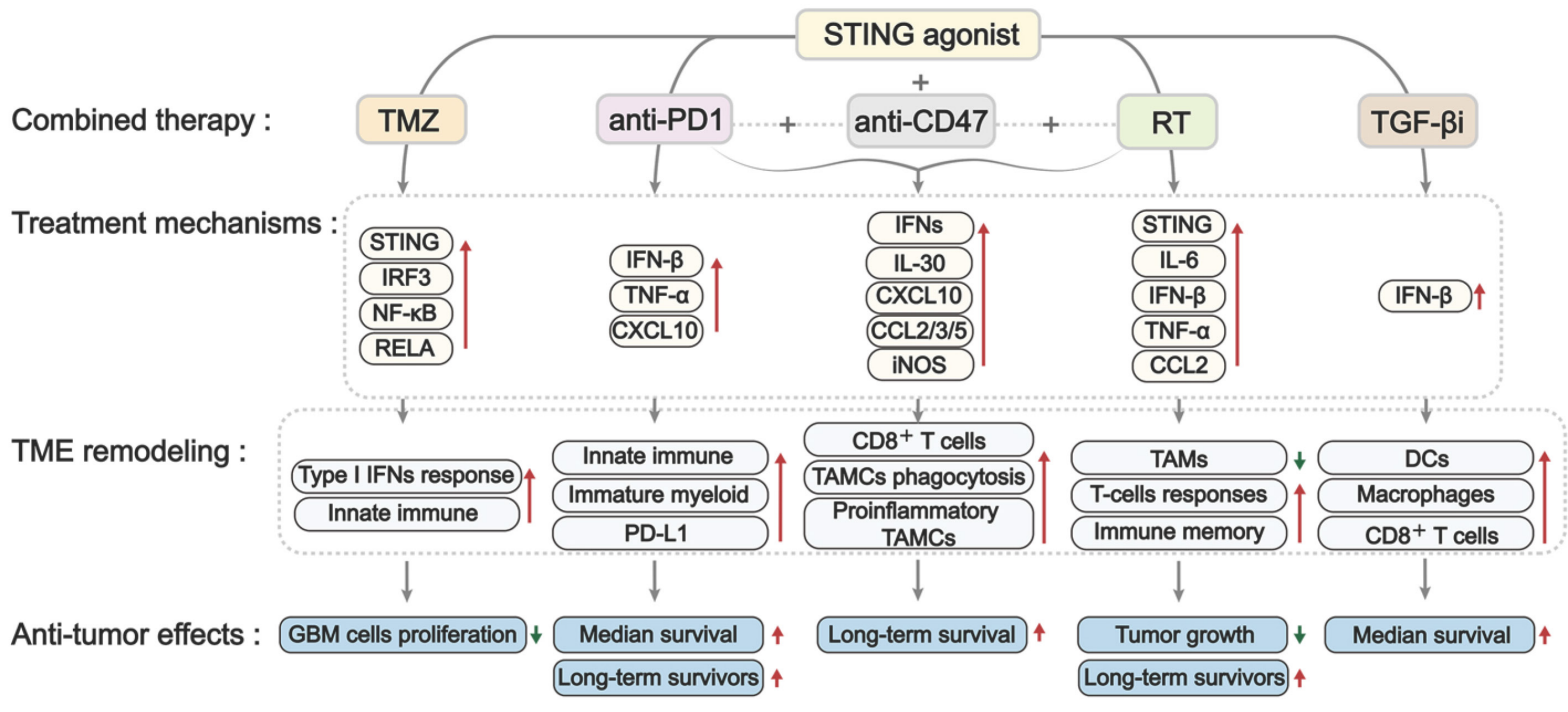
**The mechanism of combination treatment with STING agonists in GBM.** Combining STING agonists with other treatments, such as TMZ, RT, TGF-βi, anti-PD1, and anti-CD47, shows synergistic antitumor effects in GBM. This synergy is mediated through a reinforced type I IFN/cytokine response, leading to broad immune activation, including the recruitment of DCs and macrophages, improved antigen presentation, TAM reprogramming toward inflammatory and phagocytic states, and CD8⁺ T cell priming. These changes enhance antitumor immunity, inhibit tumor growth, and improve survival. TMZ: temozolomide; RT: radiotherapy; TGF-βi: TGF-β inhibitor; anti-PD1: anti-PD-1 antibody; anti-CD47: anti-CD47 antibody; TAMC(s): tumor-associated myeloid Cell(s); DC(s): dendritic cell(s); TAMs: tumor-associated macrophages.

The standard treatments for GBM include surgery, radiotherapy, and TMZ-based chemotherapy. However, due to the high genetic and molecular heterogeneity in GBM, as well as resistant mechanisms, these therapies exhibited limited success and resulted in fast tumor progression and relapse after treatment. Recently, new strategies, such as immunotherapy and small-molecule inhibitors, have shown promise in enhancing therapeutic efficacy against GBM; however, standard therapies for primary or recurrent GBM are still lacking. There has been an increased emphasis on combination therapies, leading to the proposal of various innovative strategies that synergize to treat GBM 125, 126. STING agonists showed encouraging efficacy in preclinical studies, thereby indicating their potential for combination with other approaches in GBM treatment. Here, we summarize studies on the effectiveness of various STING agonists in combination with TMZ-based chemotherapy, radiotherapy, and immunotherapy in GBM treatment (Figures 7 and Table 3).

### 6.1. STING agonist combined with TMZ-based chemotherapy in GBM

TMZ is the first-line chemotherapeutic agent for newly diagnosed GBM, primarily by inducing DNA damage. However, DNA repair pathways and the intrinsic resistance mechanisms limit the efficacy of TMZ as a monotherapy, encouraging TMZ-based combination strategies. As TMZ can trigger DNA damage-mediated dsDNA accumulation and promote the release of cGAMPs from tumor cells, it has the potential to activate the cGAS-STING signaling pathway. As expected, TMZ was demonstrated to induce IRF3 phosphorylation in APCs and promote CD45^+^ immune cell infiltration in the GL261 orthotopic mouse model 127. These findings encourage the potential combination of TMZ and STING agonists for treating GBM. As expected, 2'3'-c-di-AM(PS)₂ (Rp, Rp) (also known as ADU-S100) exhibited synergistic activity with TMZ in GBM PTEN-harboring T98G cells cells 128. A similar synergy has also been observed in melanoma, where TMZ-induced DNA damage enhanced Mn²⁺-activated cGAS-STING activation 129.

Interestingly, this combination regimen is controversial in certain studies. Yildirim *et al.* reported that this combinatorial benefit was absent in PTEN-deficient U118 GBM cells. It might be attributed to PTEN's critical role in facilitating IRF3 nuclear import 128, 130. Additionally, TMZ failed to trigger STING activation in the GL261 tumors implanted in STING-deficient mice 127. These results indicate that the synergy between TMZ and STING agonists requires an intact STING signaling pathway. Cellular stress caused by TMZ results in increased expression of GBP3, which helps stabilize STING by inhibiting its degradation. This prolonged activation of the STING pathway subsequently triggers the transcription factor NRF2, which promotes the expression of the DNA repair enzyme MGMT, aiding in the repair of DNA damage induced by TMZ. In contrast, reducing GBP3 levels destabilizes STING and restores TMZ sensitivity in GBM models. Based on these data, we conclude that persistent, uncontrolled STING activation may induce TMZ resistance 131. These data suggest a potentially pro-tumorigenic role for STING in GBM and underscore the need for molecularly precise combination strategies.

Collectively, these findings highlight both the therapeutic potential and biological complexity of combining STING agonists with TMZ in GBM (Figure 7 and Table 3). Future efforts should focus on patient stratification to determine those most likely to benefit from this combination therapy.

### 6.2. STING agonist combined with radiotherapy in GBM

Radiotherapy in conjunction with chemotherapy is the standard approach following surgery for GBM treatment. Beyond its cytotoxic role, radiotherapy can activate the cGAS-STING signaling pathway by causing DNA breaks, genomic instability, and cytosolic DNA release. Thus, there is potential for a synergistic combination of radiotherapy with STING agonists in GBM treatment. To support this notion, 3 Gy of ionizing radiation successfully induced the phosphorylation of STING and the secretion of IFN-β in mouse and human pHGG cells *in vitro*. In line with this, the combination of STING agonist with radiotherapy increased T-cell infiltration, enhanced anti-tumor effects, reduced tumor growth, and prolonged survival in the orthotopic GL261 glioma model 46. Adding STING agonist diABZI to radiotherapy resulted in approximately 60% long-term survivors by establishing antitumoral immunological memory 115.

Furthermore, radioresistance also contributes to suppression of the STING signaling pathway in GBM. The radioresistance in GBM cells is closely associated with impaired STING signaling pathway. Mechanistically, resistant cells upregulate the Golgi phosphoprotein 3-like (GOLPH3L), which interacts with STING in the Golgi after radiotherapy, driving retrograde transport of STING from the Golgi to the ER. This process suppresses the radiotherapy-induced NLRP3 (NOD-like receptor pyrin domain-containing protein 3)-driven pyroptosis 132. These observations indicate that STING agonists could potentially enhance the inherent radiosensitivity of GBM cells by promoting radiotherapy-induced pyroptosis. Taken together, the combination of STING agonists with radiotherapy represents a promising strategy to achieve dual "radiosensitization" and "immunostimulation" effects for the treatment of GBM, warranting further investigation (Figure 7 and Table 3).

### 6.3. STING agonists combined with immunotherapy in GBM

Immunotherapies, including immune checkpoint inhibitors (ICIs), cancer vaccines, oncolytic viruses, monoclonal antibodies, and T-cell-based therapies, have yielded positive outcomes in various tumors. However, their efficacy is hindered in GBM due to low infiltration of effector CD8⁺ T cells but high infiltration of immunosuppressive cells 133. Given the role of STING activation in reprogramming TME, recent strategies have explored the potential of STING agonists to increase immunotherapy efficacy in GBM. The combination of STING agonists with other immunotherapies, including TGF-β inhibitors, anti-PD-1/PD-L1 antibodies, and anti-CD47 antibodies, is summarized here (Figure 7 and Table 3).

#### 6.3.1 Combined with TGF-β inhibitors

TGF-β is a key immunosuppressive cytokine that dampens the activity and infiltration of cytotoxic immune cells (e.g., T cells and NK cells), contributing to the immune evasion 79, 134. TGF-β inhibitor is an attractive strategy to counteract immunosuppression and improve outcomes in GBM. However, due to modest single-agent efficacy, limited BBB penetration, and systemic toxicities, the therapeutic efficacy of TGF-β inhibitors is unfavorable 135, 136. Recent studies have found a synergistic effect when combining STING agonist with TGF-β inhibitor to improve TME and enhance immune activation (Figure 7 and Table 3). In orthotopic GL261 glioma models, systemically delivered cdGMP-loaded immuno-MSNs increased intratumoral macrophage and DC recruitment and elevated circulating CD8⁺ T cells. Notably, the combination of cdGMP and the TGF-β receptor 1 (TGF-βR1) kinase inhibitor Galunisertib resulted in a more prolonged median survival than untreated tumor controls 108. These data support the combination of STING agonists with TGF-β inhibitors as a promising approach to convert “cold” GBM into an immune-responsive state, harnessing the benefits of immune activation.

#### 6.3.2 Combined with anti-PD-L1/PD-1 treatment

Immune cell activity can be inhibited when PD-1 on immune cells interacts with its ligand PD-L1 on tumor cells, creating a therapeutic opportunity for PD-1/PD-L1 checkpoint inhibitors. These inhibitors have shown success in various cancers, while their efficacy in GBM has been more challenging due to the profoundly immunosuppressive TME. Recently, STING agonists have been considered a rational strategy to sensitize GBM to checkpoint inhibition by remodeling TME (Figure 7 and Table 3). In mice bearing GBM GL261 or CT-2A tumor models, the STING agonist ADU-S100 modified the tumor immune landscape, with increased infiltration of inflammatory macrophages, NK cells, and neutrophils, thereby enhancing anti-PD1 therapy and extending survival 5. Similarly, ZnCDA overcame resistance to anti-PD-L1 treatment in an orthotopic GL261 model by reversing M2-like TAM polarization to an anti-tumor M1 phenotype 46. These results highlight the potential role of STING agonists in sensitizing GBM cells to PD-1/PD-L1 inhibitors by converting an immune-cold GBM into a more inflamed, checkpoint-responsive state. Further investigation is encouraged to evaluate the efficacy of STING agonists in conjunction with other checkpoint inhibitors like CTLA-4 blockade.

#### 6.3.3 Combined with anti-CD47 antibody

CD47 is a transmembrane protein frequently overexpressed in GBM that delivers a “don't eat me” signal to macrophages by binding to signal regulatory protein α (SIRPα), thereby inhibiting macrophage-mediated tumor phagocytosis. Notably, anti-tumor therapies like radiotherapy can further upregulate CD47 expression, thereby reducing therapeutic efficacy. These observations have motivated the development of anti-CD47 monotherapies and combination approaches for treating GBM 137, 138. In GBM CT-2A preclinical models, combining a STING agonist diABZI with antibodies targeting CD47 and PD-L1 produced a more robust antitumor response than any single agent alone by enhancing myeloid cell-mediated phagocytosis 48. In line with this, engineered exosomes (BafA1@Rexo-SC) that co-deliver phosphorylated STING protein (pSTING) and CD47 nanoantibodies, activated the cGAS-STING signaling pathway in TAMs, increased the production of pro-inflammatory cytokines (IFN-α, IFN-β, and TNF-α), improved the recruitment and activation of cytotoxic T cells, and enhanced macrophage-mediated clearance of glioma cells 47 (Figure 7 and Table 3). These findings underscore a mechanistic synergy between the STING signaling pathway and CD47 blockade. Their combination is a rational and potent immunotherapeutic strategy against GBM.

Collectively, GBM presents resistance to current therapeutic approaches, primarily due to its inherent characteristics and the profoundly immunosuppressive TME. Approaches such as combining STING agonists with other therapies are promising for converting immunologically “cold” GBM into a treatment-responsive state, thereby overcoming resistance mechanisms and improving outcomes.

## 7. Challenges and limitations of STING agonists in GBM

### 7.1 STING-related neurotoxicity and pro-tumorigenic inflammation

#### 7.1.1 Neurotoxicity

A unique challenge in treating GBM is the vulnerability of the CNS. Recent studies have revealed that persistent activation of cGAS-STING in CNS-normal cells can cause chronic neuroinflammation, neurodegeneration, and cognitive decline in ageing mouse models. Specifically, in microglia, cytosolic mitochondrial DNA triggers cGAS-STING signaling, leading to sustained production of type I IFNs, TNF-α, CXCL10, and CCL5, which ultimately induce neuronal toxicity and memory impairment 139, 140. These findings raise a serious concern that STING agonists might exacerbate neurotoxic effects after prolonged exposure. Moreover, other STING epigenetic regulators may also contribute to toxicity. Hypermethylation of the STING promoter, which accounts for STING silencing in neoplastic cells, is also present in normal brains 23. Thus, when systemic DNMT inhibition restores STING expression in tumors, it may concurrently trigger STING-mediated toxicity in non-malignant neural cells. Although such on-target toxicity has not been reported in GBM models, its risk remains plausible and warrants careful evaluation. Collectively, these data emphasize the narrow therapeutic window of STING activation in the brain. Safe and effective translation will demand the development of cautious dosing protocols and tumor-targeted delivery systems to minimize off-tumor toxicity.

#### 7.1.2 Pro-tumorigenic inflammation

Persistent cGAS-STING signaling activation establishes a chronic inflammatory state, with sustained IL-6 release being a key component. IL-6 then activates the STAT3 pathway, which upregulates immune-checkpoint molecules (e.g., PD-L1), skews the TME toward an immunosuppressive state 141, 142, and maintains cancer stemness, thereby fostering tumor progression rather than rejection 143. To address this, optimized pulse-dosing regimens and rational combinations, such as targeting IL-6-STAT3, may be needed.

### 7.2 Phasic dosing of STING agonists

STING activation induces early, mid, and late immune phases, and each phase has different therapeutic implications. Typically, type I IFNs and chemokines peak in the early phase, followed by the recruitment and expansion of effector T cells in the mid-phase. Under prolonged or chronic stimulation, however, late-phase responses may shift toward protumorigenic inflammatory programs. These temporal dynamics suggest that pulse-like, intermittent dosing schedules, rather than continuous or densely repeated dosing, might better sustain antitumour immunity while limiting chronic toxicity 144, 145.

### 7.3 GBM heterogeneity and biomarker selection

The molecular heterogeneity of GBM contributes to variable responses to STING agonists. Thus, the molecular stratification and biomarker-based patient selection are urgently needed. As reported, GBM with silenced STING, due to promoter hypermethylation (cg16983159), are typically refractory to agonist therapy 23. This result indicates that the response to agonists is intrinsic STING-dependent. Therefore, assessing intrinsic STING expression with epigenetic marks could serve as a biomarker to identify likely responders. Beyond epigenetic regulation, specific genomic context also modulates STING. In HGG harboring wild-type ATRX and IDH1, ATRX expression positively correlates with STING expression. It seems that ATRX may serve as another stratification marker 146. Meanwhile, specific genetic alterations might predict poor combinatory outcomes. For example, PTEN loss is associated with a diminished synergistic effect between STING activation and TMZ 128. On the contrary, deficient DNA repair capacity can define a subset as a STING-hyperresponsive and favourable response in pHGG models harbouring H3.3-G34 mutations 115.

### 7.4 Delivery-system limitations and translational barriers

Current delivery platforms for STING agonists still face translational obstacles. Increasing consideration is focused on poor brain distribution, systemic toxicity, manufacturability, and stability issues 144. Firstly, achieving therapeutic concentrations in the brain tumor is difficult. Locally administered nanoparticles are clinically cumbersome, whereas systemic BBB-targeted strategies still result in incomplete and heterogeneous tumor distribution 102, 147. Secondly, encapsulated STING agonists can still cause systemic inflammatory toxicity and the off-target accumulation in organs such as the liver and spleen, as observed in non-GBM studies. This brings a risk, particularly in frail GBM patients 144, 148. Thirdly, many of the most efficacious carriers rely on multi-component polymers, proteins, or cell-membrane coatings, which complicates large-scale manufacturing, reproducibility, and long-term storage stability. Finally, both CDN-based STING agonists and several nanocarriers show suboptimal physicochemical stability and rapid degradation. Specialized formulations or cold-chain logistics are often required, further increasing the practical barriers to widespread clinical implementation 149.

## 8. Summary and prospects

Accumulating evidence highlights the central role of the STING signaling pathway in activating antitumor immunity in GBM. By driving type I IFN induction, the STING signaling pathway recruits innate immune cells, promotes their differentiation and maturation, enhances antigen presentation, and connects innate immune sensing to adaptive immune activation. Many preclinical studies indicate that STING agonists can convert immunologically “cold” glioma into “hot” tumors and thus improve survival. Hence, STING agonists represent a novel strategy for GBM. Combination approaches that integrate STING agonists with radiotherapy, chemotherapy, or other immune therapies are particularly encouraged. However, before translating STING agonists into clinical treatment, more attention should be paid to elucidating the mechanisms underlying cell-specific responses, toxicity, resistance, and dosing regimen, as well as to developing and optimizing effective delivery systems.

## Figures and Tables

**Table 1:** The summary of STING agonists in GBM

STING agonist	BBB penetration	Animals	Therapeutic dose	Development stage	Main effects in GBM	References
CDN Agonists						
c-di-GMP	Limited	GL261 Orthotopic model (C57BL/6)	c-di-GMP: 4μg /2 µL (i.c.) single injection; cdGMP-loaded immuno-MSNs (10 μg of cdGMP): (i.v.) on days 7, 8 and 14 post tumor inoculation	Preclinical	Induces type I IFN response; Increases inflammatory cytokines secretion; Promotes DCs and macrophages recruitment; Enhances antigen presentation; Enhances CD8⁺ T cell infiltration; Promotes activation, proliferation, differentiation of CTLs; Improves survival in GL261 model.	108 109
ADU-S100 (MIW815)	Limited	GL261/CT-2A Orthotopic model (C57BL/6)	GL261: 50 µg (i.c.) single injection; CT-2A: 50 µg (i.c.) single injection	Preclinical	Increases inflammatory response; Promotes immune cell infiltration (NK cells, macrophages); Increases T cell proportions; Remodels the TME and induces immune memory; Upregulates PD-L1; Activates innate immune response; Survival benefit.	5
IACS-8779	Limited	Spontaneous intracranial GBM (canine)	5/10/15/20µg (i.c.) every 4-6 weeks (≥2 cycles); Maximum tolerated: 15µg	Preclinical	Induces radiographic tumor regression; Median PFS of 14 weeks and median OS of 32 weeks in preclinical model.	112
IACS-8803	Limited	GL261/CT-2A/QPP4/QPP8 orthotopic (C57BL/6J) U87 orthotopic (huNOG-EXL humanized)	2.5/5 µg (i.c.) GL261 (day 7, 14); CT-2A (day 7, 17); QPP4 (day 14, 28); QPP8 (day 14, 21, 28); U87 (day 5, 10, 15)	Preclinical	Induces myeloid-mediated TME reprogramming; Enhances antigen presentation; Decreases immunosuppressive markers in myeloid; Enhances CD8^+^ T cell and NK effector responses; Improves survival across models.	19
Non-Nucleotide Agonists						
diABZI	Limited	H3.3-G34R pHGG Orthotopic model CT-2A Orthotopic model (C57BL/6)	5 µg/4 µL (i.c.) on days 13 and 19 0.25 mg/kg(i.c., diABZI loaded B-LNP) on day 14 2.5 mg/kg(i.v., diABZI loaded P-LNP) on day 14	Preclinical	Induces DNA repair deficiency and cGAS-STING activation; Switches transcription and metabolism of TAMCs; Enhances antigen presentation; Reprograms TAMCs to a pro-inflammatory state; Promotes phagocytosis; Enhance CD8⁺ T cell immunity; Synergizes with RT to establish long-term memory.	115 48
SR717	Limited	GL261 orthotopic Model (C57BL/6)	SR717@RGE-HFn: 5 mg/kg(i.v.) every 3 days (5 times in total)	Preclinical	Enhances STING downstream signaling *in vitro*; Elicits local innate immune response; Increases proinflammatory cytokines; Recruitment of CD8+ T cells, NK cells and DCs; Reduces tumor growth by ~ 55%; Improves physical status; Without systemic toxicity; Extends the median survival.	7 116
ASA404 (DMXAA)	Limited	U87/LN229/U251/LN308 subcutaneous model U87/U251 Orthotopic model (athymic nude mice) Tu-2449 Orthotopic model (B6C3F1)	U87/LN229/U251/LN308: 25 mg/kg(i.p.) single injection or twice weekly from day 25 to day 60 U87/U251: 25 mg/kg(i.p.) single injection or twice weekly from day 7 Tu-2449: 5 mg/kg(i.p.) single injection	Preclinical	Shows anti-tumor effects in subcutaneous glioma models; Fails in orthotopic glioma, brain metastasis, and malignant meningioma models.	118

Abbreviations: i.v.: intravenous; i.p.: intraperitoneal; i.c.: intracranial; RT: radiotherapy; GBM: glioblastoma; CTL: cytotoxic T lymphocytes; immuno-MSNs: immunostimulatory mesoporous silica nanoparticle; PFS: Progression-Free Survival; OS: Overall Survival; LNP: lipid nanoparticle; B-LNP: bridging-lipid nanoparticle; P-LNP: LNP with anti-PD-L1 functionalization; TAMCs: tumor-associated myeloid cells; huNOG-EXL humanized: humanized NOG (NOD/Shi-scid/IL-2Rγ^null^ ) with expanded leukocytes

**Table 2:** The summary of innovative delivery platforms

Platforms	Composition	Main advantage	Main effects	Reference
DT-Exo-STING (dendritic cell-tumor hybrid exosome)	Dendritic cell + tumor hybrid cell-derived chimeric exosomes + cdGAMP	Dual T-cell activation via broad tumor antigens; Enhances BBB penetration; Enhances antigen presentation via cytosolic delivery of STING agonists.	Increases tumor-specific T-cell immunoresponse; Reprograms TME toward inflammation; Eradicates primary or residual intracranial tumors; Improves sensitivity to ICB; Induces systemic immune memory.	119
bALG@DT-Exo-STING (artificial-LN exosome gel)	Alginate polymers + Dendritic cell/tumor hybrid cell-derived chimeric exosomes + cdGMP-Dy547	Activates the tumor-infiltrating T cells directly; Avoids clearance of compound by the immune system.	Induces durable T-cell immunity; Eliminates residual lesions in postsurgical GBM model; Reduces recurrence risk.	120
SRHLS (sequential release hydroLipo system)	Carboxyethyl chitosan-Oxidized sodium alginate hydrogel + Decitabine + diABZI	Restores STING then sustains activation (sequential); Achieves epigenetic reprogramming.	Remodels the TME; Reprograms TAMs towards M1-like; Enhances antitumor immunity; Inhibits tumor growth; Reduces recurrence and metastasis.	121
ZnCDA (zinc phosphate NCP loaded with CDA)	Zn-phosphate NCP + PEGylated lipid bilayer + CDA	Ensures stability and controlled release of CDA; Provides pharmacokinetic advantages; Prolongs CDA circulation.	Repolarizes TAMs from M2- to M1-like phenotype; Suppresses primary/metastatic tumors; Synergy with ICB/RT.	46
PLHM-DOX NPs	A copolymer comprising PEG^5000^, seven lysines, eight histidines (PLH)-Mn²⁺ + DOX	Mn²⁺ activates cGAS-STING; type I IFN production.	Promotes DC maturation and CD8⁺ T-cell infiltration; Synergy with DOX; Strong tumor suppression.	122
TCe6@Cu/TP5 NPs	Ce6-TPP + Cu²⁺ + TP5	Enhances BBB crossing via copper transport mechanism; Cuproptosis activates AMPK pathway-mediatd PD-L1 degradation; Endogenous cGAS-STING activation.	Promotes the proliferation and differentiation of DCs and T cells; Synergies with photoimmunotherapy to further enhances antitumor immunity.	68
BM@MnP-BSA-aPD-1	Microglia-membrane coating + MnP + anti- PD-1 antibody	Biomimetic BBB translocation and TME targeting; Integrates metallo-immuno-therapy with PTT; Activates the STING pathway.	Induces ICD; Reprograms immunosuppressive TME; Synergies with photothermal-immunotherapy.	123
NED@MnO₂-DOX	Vitamin D₃-lipid hybrid neutrophil membrane + MnO₂ + DOX	Activates cGAS-STING; Increases IFN-β and pro-inflammatory cytokines.	Induces DC maturation; Enhances CD8⁺ T-cell response; Converts cold TME to hot.	124

Abbreviations: ICB: Immune Checkpoint Blockade; bALG: branched alginic acid; NCP: nanoscale coordination polymer; CDA: cyclic dimeric adenosine monophosphate; PLHM: PEG^5000,^ seven lysines, and eight histidines chelated Mn^2+^; DOX: doxorubicin; NPs: nanoparticles; Ce6: Chlorin e6; TPP: triphenylphosphorus; TP5: thymopentin; BM: BV2 microglia membrane; MnP: manganese porphyrin nanoparticles; BSA: bovine serum albumin; ICD: immunogenic cell death

**Table 3:** The summary of combination therapies with STING agonist in GBM

Combination		Animal, Cell	Therapeutic regimen	Mechanism	Effects	References
TMZ						
TMZ+ ADU-S100		T98G (PTEN-harboring)/U118MG (PTEN-deficient)	*In vitro*: 2 µg/mL ADU-S100 + TMZ (600 µM for T98G; 400 µM for U118MG)	T98G: increases STING, IRF3, NF-κB, and RELA mRNA expression; Elevates STING and NF-κB proteins.	T98G: Combination > TMZ alone (enhanced response); U118MG: No added benefit over TMZ alone; Suggests biomarker-guided selection (PTEN).	128
RT						
RT+ diABZl		H3.3-G34R pHGGOrthotopic model(C57BL/6)	RT: 2 Gy/day for 10 days (total 20 Gy); diABZI: 5µg (i.c.), twice on days 13 and 19 post-implantation	RT-induced DNA damage triggers cGAS/STING signaling; diABZl enhances STING-mediated antitumor immunity.	Improves survival with ~60% long-term survivors; Establishes immunological memory upon tumor rechallenge.	115
RT+ ZnCDA		GL261 Orthotopic model(C57BL/6)	RT: 3 × 2 Gy at day 8, day 11, day 14 post-tumor implantation ZnCDA: 10 μg (i.v.), once weekly for three doses	NCP-based ZnCDA accumulates in tumors and preferentially targets TAMs; Primes anti-tumor T-cell responses.	Increases T-cell infiltration in GL261; Suppresses tumor growth; Prolongs survival in GL261 model.	46
Immunotherapy						
TGF-β inhibitor	Galunisertib + cdGMP	GL261 Orthotopic model(C57BL/6)	Galunisertib (2.5 mg/kg i.p., 5 days/week from day 3) + cdGMP-loaded MSN (10 μg cdGMP i.v. on days 7,8, and 14)	TGF-β pathway inhibition and STING activation.	Promotes DC and macrophage recruitment; Enhances CD8⁺ T cell activity; Strengthens antitumor immunity; Improves survival.	108
anti-PD-1	anti-PD-1 + ADU-S100	CT-2A Orthotopic model(C57BL/6)	CT-2A model: ADU-S100 (35 μg) + anti-PD-1 antibody (25 or 50 μg) loaded hydrogel (i.c.) on day 7	STING agonist induces rapid innate immune activation; Delays T cell exhaustion; Counteracts chronic immunosuppression from STING activation.	Combination induces long-term survivors; Enhances therapeutic efficacy compared to ADU-S100 alone.	5
anti-CD47	B-LNP (αPD-L1/αCD47-decorated) + diABZI+RT	CT-2A Orthotopic model(C57BL/6)	0.25 mg diABZI/kg (i.c.); anti-PD-L1 antibody and anti-CD47 antibody conjugated on B-LNP surface (lipid:antibody = 8.7:1 (w/w)); RT: 3 Gy/day × 3 days	Activates STING pathway in TAMCs; Immune checkpoint blockade; Enhances antitumor immunity.	Enhances CD8+ T cell infiltration and activation; Reduces tumor burden; Increases TAMC phagocytic activity of glioma; Improves survival including long-term survivors.	48

Abbreviations: RT: radiotherapy; TMZ: temozolomide; MSN: mesoporous silica nanoparticle; w/w: weight/weight; i.v.: intravenous; i.p.: intraperitoneal; i.c.: intracranial; aPD-1: anti-PD-1 antibody; aPD-L1: anti-PD-L1 antibody; aCD47: anti-CD47 antibody; TAMC(s): tumor-associated myeloid Cell(s); DC(s): dendritic cell(s); NK cell(s): natural killer cell(s)

## Abbreviations

GBM: glioblastoma
TME: tumor microenvironment
STING: stimulator of interferon genes
IFN: interferon
CNS: central nervous system
WHO: World Health Organization
TMZ: temozolomide
dsDNA: double-stranded DNA
cGAS: cyclic GMP-AMP synthase
MDSCs: myeloid-derived suppressor cells
DC: dendritic cell
ER: endoplasmic reticulum
TBK1: TANK-binding kinase 1
IRF3: interferon regulatory factor 3
ISGs: IFN-stimulated genes
IKK complex: IκB kinase complex
PERK: protein kinase RNA-like ER kinase
eIF2α: eukaryotic initiation factor 2α
PP2A: protein phosphatase 2A
STRN4: striatin 4
MST1/2: mammalian STE20-like protein kinase1/2
YAP: Yes-associated protein
TAZ: Transcriptional co-activator with PDZ-binding motif
DNMT: DNA methyltransferase
DDR: DNA damage response
ATM: ataxia telangiectasia mutated
USP7: ubiquitin-specific peptidase 7
RPA1: replication protein A1
ATR: Ataxia Telangiectasia and Rad3-related
APCs: antigen-presenting cells
TAMs: tumor-associated macrophages
EVs: extracellular vesicles
BBB: blood-brain barrier
Tregs: regulatory T cells
ARG1: arginase 1
M-MDSCs: monocytic-MDSCs
TAMCs: tumor-associated myeloid cells
TANs: tumor-associated neutrophils
DAMPs: danger-associated molecular patterns
NETs: neutrophil extracellular traps
TLS: tertiary lymphoid structure
NK: natural killer
Bregs: regulatory B cells
NRF2: nuclear factor erythroid 2-related factor 2
CDNs: cyclic dinucleotides
pHGG: pediatric high-grade glioma
GOLPH3L: Golgi phosphoprotein 3-like
ICIs: immune checkpoint inhibitors
SIRPα: signal regulatory protein α
pSTING: phosphorylated STING
CTL: cytotoxic T lymphocytes
PFS: Progression-Free Survival
OS: Overall Survival
LNP: lipid nanoparticle
B-LNP: bridging-lipid nanoparticle
P-LNP: LNP with anti-PD-L1 functionalization
ICB: Immune Checkpoint Blockade
bALG: branched alginic acid
NCP: nanoscale coordination polymer
CDA: cyclic dimeric adenosine monophosphate
DOX: doxorubicin
NPs: nanoparticles
Ce6: Chlorin e6
TPP: triphenylphosphorus
TP5: thymopentin
BM: BV2 microglia membrane
MnP: manganese porphyrin nanoparticles
BSA: bovine serum albumin
ICD: immunogenic cell death
PD-1: programmed cell death protein 1
PD-L1: programmed death-ligand 1
aPD-1: anti-PD-1 antibody
aPD-L1: anti-PD-L1 antibody
aCD47: anti-CD47 antibody
